# Genome-wide regulatory dynamics of translation in the *Plasmodium falciparum* asexual blood stages

**DOI:** 10.7554/eLife.04106

**Published:** 2014-12-10

**Authors:** Florence Caro, Vida Ahyong, Miguel Betegon, Joseph L DeRisi

**Affiliations:** 1 Department of Biochemistry and Biophysics, University of California, San Francisco, San Francisco, United States; 2 Howard Hughes Medical Institute, University of California, San Francisco, San Francisco, United States; Cold Spring Harbor Laboratory, United States

**Keywords:** *Plasmodium falciparum*, translation, ribosome profiling, malaria, other

## Abstract

The characterization of the transcriptome and proteome of *Plasmodium falciparum* has been a tremendous resource for the understanding of the molecular physiology of this parasite. However, the translational dynamics that link steady-state mRNA with protein levels are not well understood. In this study, we bridge this disconnect by measuring genome-wide translation using ribosome profiling, through five stages of the *P. falciparum* blood phase developmental cycle. Our findings show that transcription and translation are tightly coupled, with overt translational control occurring for less than 10% of the transcriptome. Translationally regulated genes are predominantly associated with merozoite egress functions. We systematically define mRNA 5′ leader sequences, and 3′ UTRs, as well as antisense transcripts, along with ribosome occupancy for each, and establish that accumulation of ribosomes on 5′ leaders is a common transcript feature. This work represents the highest resolution and broadest portrait of gene expression and translation to date for this medically important parasite.

**DOI:** http://dx.doi.org/10.7554/eLife.04106.001

## eLife digest

The genome of an organism includes all of the genes or information necessary to build, maintain, and replicate that organism. However, cells with the same genome—such as a skin cell and a liver cell from the same person—can look and behave very differently depending on which of the genes in their genomes they express, and to what extent.

For a gene to be expressed, its DNA is ‘transcribed’ to make an RNA molecule, which is then ‘translated’ to make a protein. Efforts to measure the transcription and translation processes in diseased cells, or in the microorganisms that cause infections, may lead to new treatments and preventative medicines. Such work is currently ongoing in the global effort to treat and prevent malaria.

Malaria is both preventable and curable, yet over 600,000 people are estimated to die from this disease each year. The disease is caused by a single-celled parasite called *Plasmodium*. Mosquitoes carry the parasites in their salivary glands, and when a mosquito bites a human, these parasites are injected into the bloodstream with the mosquito's saliva. *Plasmodium* parasites then travel to and infect the liver, before bursting out of this tissue into the bloodstream. Here, the parasites infect red blood cells and undergo rounds of replication during which the symptoms of the disease are manifested. It is also during this bloodstream phase that parasites can develop into forms capable of infecting another mosquito and continuing the transmission cycle.

The genes, RNA molecules, and proteins of the *Plasmodium falciparum* parasite—which causes the most serious cases of malaria in humans—have been cataloged to better understand the biology of this parasite. However, the processes that control how, and when, an RNA transcript is translated into a protein are not well understood.

Now Caro et al. have uncovered which RNA molecules are being translated, and by how much, during *Plasmodium* development within the blood. The transcription and translation of genes in this parasite were found to be tightly linked processes; the expression of only a few genes was controlled more by the translation process than by transcription. These translationally regulated genes were found mainly to be those that encode proteins involved in the parasite's exit from the red blood cells and spread throughout the bloodstream.

Caro et al. discovered that genetic regulation of the malaria parasite resembles a preset genetic program, rather than a system that responds to changes and external signals. As such, these findings suggest that targeting such a genetic program within *Plasmodium* and preventing its implementation could prove an effective strategy to curb the spread of malaria.

**DOI:** http://dx.doi.org/10.7554/eLife.04106.002

## Introduction

The transcriptome of the intraerythrocytic developmental cycle (IDC) of *P. falciparum* is characterized by a continuous cascade wherein the expression of the majority of genes is maximally induced once per cycle and their timing correlates well with the timing for the respective protein's biological function (Bozdech et al., 2003). The apparent lack of dynamic transcriptional regulation suggested that complementary post-transcriptional mechanisms could play an important role in the regulation of parasite gene expression (Hughes et al., 2010). This is a reasonable assumption, given that global or gene-specific translational regulation of gene expression is a mechanism that allows fast adaptations during drastic changes in environmental conditions as well as during rapid transitions in developmental programs. Indeed a few examples of translational control in *Plasmodium* have been reported. In sporozoites present in the mosquito salivary gland, phosphorylation of the eukaryotic translation initiation factor eIF2α by the kinase IK2, inhibits translation and causes accumulation of mRNAs into granules. Translational repression is alleviated by eIF2α phosphatase during the transition into the mammalian host, allowing parasites to transform into the liver stages (Zhang et al., 2010). Similarly, PK4 kinase activity leads to the reduction of global protein synthesis through phosphorylation of eIF2α in schizonts and gametocytes and is essential for the completion of the parasite's erythrocytic cycle (Zhang et al., 2012). Gene-specific translational regulation has also been observed in *P. falciparum* and is mediated by cis-acting sequences in combination with RNA-binding proteins. For example, dihydrofolate reductase-thymidylate synthase (DHFR-TS) binds within the coding region of its own cognate mRNA to repress translation (Zhang and Rathod, 2002) and antifolate treatment has been shown to relieve this repressive effect without alteration of mRNA levels (Nirmalan et al., 2004a). In *Plasmodium berghei,* storage of translationally repressed mRNAs prior to fertilization is mediated by mRNA binding via the RNA helicase DOZI and the Sm-like factor CITH (Mair et al., 2006, 2010). Upstream open reading frames (uORFs) found on 5′ UTRs of transcripts have been reported to regulate the translation of specific genes (Morris and Geballe, 2000). In *P. falciparum*, the only uORF described and functionally characterized to date is a 120 codon region upstream of the *var2csa* (PFL0030c) coding region, a unique variant of the surface antigen PfEMP1 that mediates adhesion to placenta in pregnant women (Amulic et al., 2009). In this case, translation of the uORF modulates repression of var2csa translation. Aside from these examples, the extent to which global and gene-specific translational control operates in *P. falciparum* during the IDC remains sparse.

Since the *P. falciparum* genome was fully sequenced (Gardner et al., 2002), several large-scale studies have provided detailed insights into the expression of genes and proteins across the parasite's life cycle. Parallel mass spectrometry-based proteomics and genome-wide expression profiling revealed differences between mRNA abundance and the accumulation of the corresponding protein, supporting the notion that post-transcriptional regulation of gene expression is at play in this parasite (Le Roch et al., 2004; Nirmalan et al., 2004b; Foth et al., 2011). These methods, however, are limited in their ability to measure low abundance proteins and do not capture the underlying relationship between transcriptional activity and translational efficiency. More recently, polysome profiling was used to monitor discrepancies between polysome-associated and steady-state mRNAs in 30% of the *P. falciparum* blood stage transcriptome (Bunnik et al., 2013); however, this approach does not reveal the precise localization of the ribosomes, and thus can not be used to accurately assess the translational efficiency of a given mRNA (Ingolia, 2014).

Here, we adapted the ribosome profiling technique (Ingolia et al., 2009) to describe the translational dynamics of the *P. falciparum* asexual blood stage transcriptome. We simultaneously evaluate mRNA abundance, gene structure, ribosome positioning, and translational efficiency for genes expressed through five stages of the IDC. We demonstrate that the data are highly reproducible, and we find that the translational efficiency of the majority of mRNAs expressed follows a narrow distribution, exhibiting a tight coupling between transcription and translation. Only 10% of the genes expressed deviate from this trend and are translationally up- or down-regulated. We found a surprising amount of ribosome density associated with 5′ leaders of transcripts particularly in genes with functions associated with merozoite egress and invasion. Overall, the precision and depth of the dataset presented herein add significantly to our understanding of *P. falciparum* gene expression by linking transcriptional and translational dynamics throughout the blood stages.

## Results

### Overview of ribosome profiling in *P. falciparum* asexual blood stages

**Figure 1.**
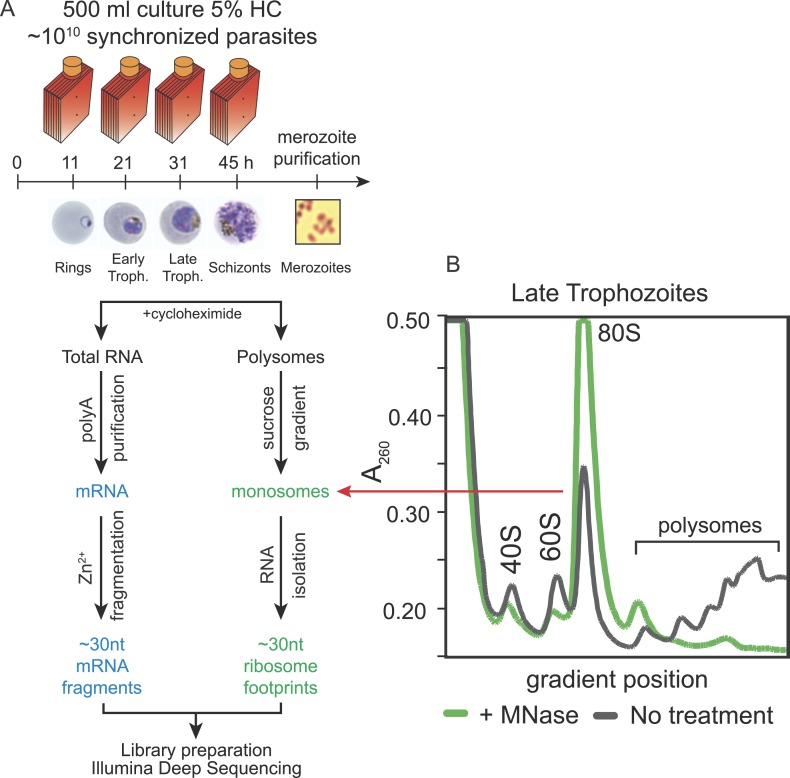
Ribosome profiling of the *P. falciparum* asexual blood stages, experimental outline. (**A**) Synchronized parasite cultures were maintained in hyperflasks at 5% hematocrit and maximum 15% parasitemia. Cycloheximide-treated cultures containing ∼10^10^ parasites were harvested at ring, early trophozoite, late trophozoite and schizont stages (11, 21, 31, and 45 hpi, respectively) for total RNA or polysome isolation. Merozoites were purified through magnetic isolation of late stage schizonts (see ‘Materials and methods’). Nuclease treated polysomes were fractionated on a sucrose gradient. Ribosome footprints (∼30 nt) derived from the monosome peak (dashed red line) or chemically fragmented polyA purified mRNA (∼30 nt) were used to build sequencing libraries. mRNA and ribosome footprint samples were processed in parallel to create deep sequencing libraries compatible with the Illumina platform. (**B**) Sucrose gradient A260 absorbance profile of polysome extracts derived from late trophozoites treated with micrococcal nuclease (green, +MNase) or untreated controls (gray, No treatment). Red arrow indicates the 80S monosome peak collected for ribosome footprint library preparation. **DOI:** http://dx.doi.org/10.7554/eLife.04106.003 10.7554/eLife.04106.004Figure 1—source data 1.Illumina sequencing mapping statistics against *P. facliparum* W2 SNP-corrected genome.**DOI:** http://dx.doi.org/10.7554/eLife.04106.004

To create whole-genome, high-resolution profiles of mRNA abundance and translation during in vivo blood stage development of *P. falciparum,* we adapted the ribosome profiling technique described by Ingolia et al. (2009). Ribosome profiling is based on the deep sequencing of ribosome protected mRNA fragments obtained by nuclease digestion of polysomes, cycloheximide-arrested ribosomes bound to mRNA. These fragments represent the exact location of the ribosome at the moment the sample was harvested. Five stages representative of the 48-hr IDC of *P. falciparum* were harvested for both mRNA and polysome isolation; ring, early trophozoite, late trophozoite, schizont stages, and purified merozoites. To assess the reproducibility of the data, we harvested independent biological replicates of each stage. Polysomes were isolated in the presence of the translation elongation inhibitor cycloheximide, then nuclease digested to produce monosomes, and sedimented by centrifugation on a sucrose gradient (Figure 1 and Figure 1—figure supplement 1). To minimize isolation of RNA fragments bound by proteins other than 80S ribosomes, RNA was extracted only from the fractions of the sucrose gradient containing the monosome peak. The resulting ∼30 nt fragments of RNA, corresponding to ribosome footprints, were processed into strand-specific deep sequencing libraries in parallel with the mRNA samples, fragmented to ∼30 nt for consistency. Despite the unusually high AT content of the *P. falciparum* genome, over 92% of all 30 nt sequenced reads, derived from coding sequences (CDSs), mapped uniquely to the genome (Figure 1—source data 1 and Figure 1—figure supplement 2).

**Figure 1—figure supplement 1.**
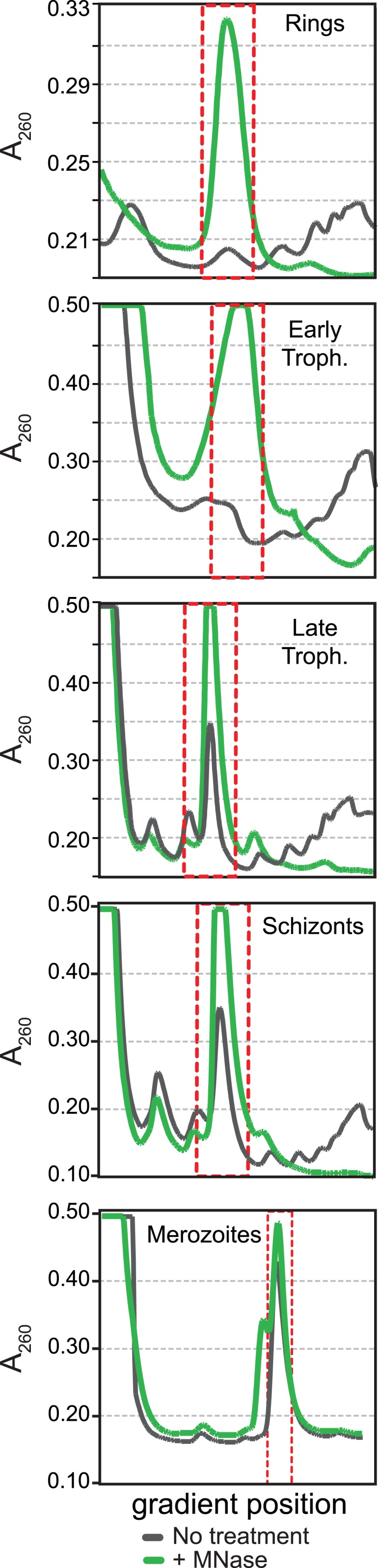
Polysome profiles of the *P. falciparum* asexual blood stages. Sucrose gradient A260 absorbance profiles of polysome extracts treated with micrococcal nuclease (green, +MNase) and untreated controls (gray, No treatment). Red dotted line indicates monosome peak harvested for ribosome footprint library generation. **DOI:** http://dx.doi.org/10.7554/eLife.04106.005

**Figure 1—figure supplement 2.**
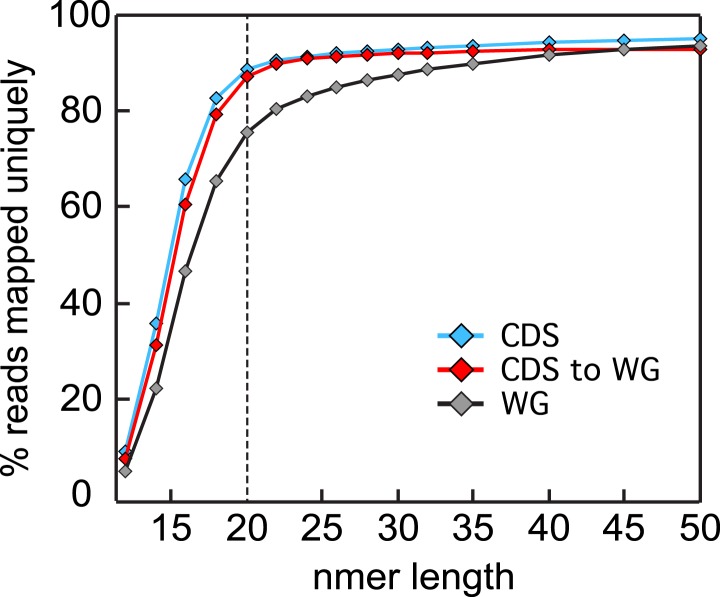
Read size influence on mappability. Single nucleotide sliding windows ranging from 10 to 50 nt were used to generate in silico libraries of the *P. falciparum* W2 SNP corrected genome. These were uniquely aligned, allowing no mismatches, to either the whole genome (gray, WG) or the coding sequences (blue, CDS) using Bowtie (Mortazavi et al., 2008) and the percentage of aligned reads were calculated for each window size. The analysis was repeated using sliding windows generated from the coding genome only (red, CDS to WG) for a more representative mappability estimate of an RNA-seq data set. Read sizes of ≥20 nt asymptotically approach maximum mappability percentages. **DOI:** http://dx.doi.org/10.7554/eLife.04106.006

**Figure 1—figure supplement 3.**
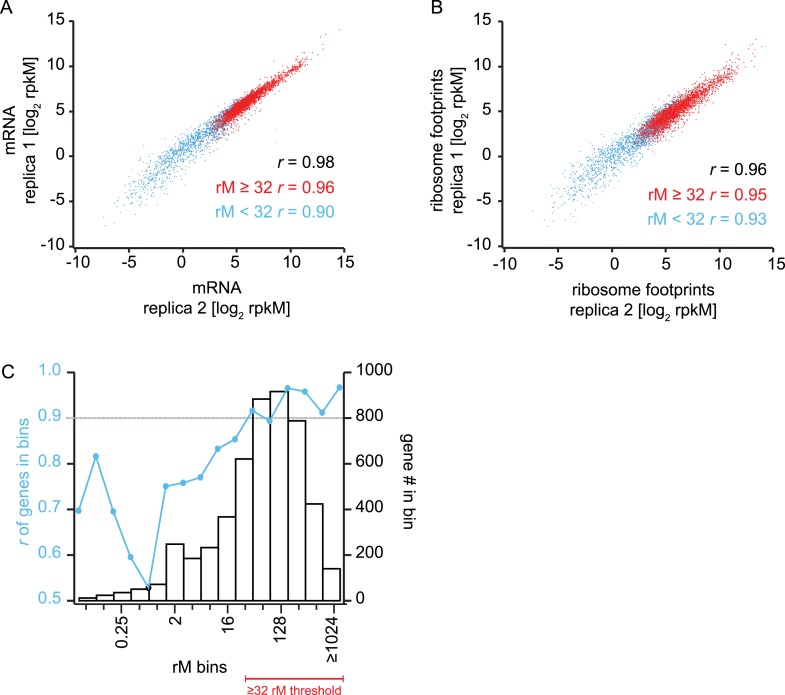
Reproducibility and coverage threshold determination using two fully independent biological replicates. mRNA abundance measurements (**A**) and ribosome footprint densities (**B**) in terms of rpkM in two fully independent biological replicates of the late trophozoite timepoint. Genes with at least ≥32 total mRNA reads counted (rM) are highly reproducible (r ≥ 0.9) across replicas (A and B red dots, and Figure D) whereas low read counts have a negative effect on rpkM reproducibility (A and B blue dots, and C). (**C**) Genes were binned based on their rM in replica 1. In each bin Pearson correlations of rpkM values of replica 1 and replica 2 were calculated. At 32 rM, *r* values were consistently above 0.9 indicating that rpkMs calculated for genes with ≥32 rM are highly reproducible across replicates, and this is independent of the number of genes in the bin. *r* = Pearson correlation coefficient. **DOI:** http://dx.doi.org/10.7554/eLife.04106.007

**Figure 2.**
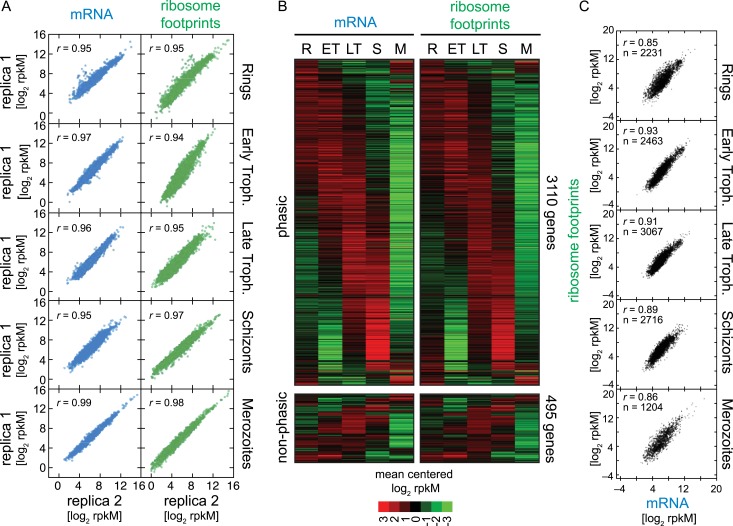
Ribosome profiling through the *P. falciparum* IDC. (**A**) Reproducibility among biological replicates. Two fully independent biological replicas of each stage were sampled for RNA-seq (left panels, blue) and ribosome profiling (right panels, green). Each dot represents the log_2_ rpkM measured for each gene in each stage. *r* = Pearson correlation coefficient. (**B**) Gene expression and translation are tightly coupled during the *P. falciparum* IDC. Phaseograms of mRNA (left heatmap) and ribosome footprint density (right heatmap) as a function of development for 3110 phasic and 495 non-phasic genes organized in the same order in the left and right heatmap. Data represent mean centered log_2_ mRNA and ribosome footprint rpkM values for each gene (rows) in each sampled stage (columns). R = rings, ET = early trophozoites, LT = late trophozoites, S = schizonts, M = merozoites. (**C**) log_2_ rpkM of mRNA abundance vs ribosome footprint density for all genes expressed (rM ≥ 32) across the IDC. Pearson correlation coefficients *r* ≥ 85. n = total number of genes. **DOI:** http://dx.doi.org/10.7554/eLife.04106.008 10.7554/eLife.04106.009Figure 2—source data 1.*P. falciparum* ribosome profiling data set.**DOI:** http://dx.doi.org/10.7554/eLife.04106.009

To quantitatively obtain mRNA abundance and ribosome footprint density measures, we calculated rpkMs (reads per kilobase of exon model per million reads mapped, as in Mortazavi et al. (2008) for each gene. We established the minimum number of mRNA reads sequenced per coding region (rM; reads per million reads mapped) required to confidently include genes in downstream analyses, to be ≥ 32 rM (‘Materials and methods’, Figure 1—figure supplement 3). Using this conservative threshold, 3605 genes qualified for further analysis. Between biological replicates, Pearson correlation values were consistently high, ranging from *r* = 0.94 to *r* = 0.99 (Figure 2A), highlighting the quality and reproducibility of our data. In addition, we compared the RNA-seq transcriptome of the five stages sampled to our previously published transcriptome data set, originally generated using long oligo microarrays (Bozdech et al., 2003). The RNA-Seq transcription profiles of the set of genes shared by the two data sets (n = 1829) were highly correlated (average *r* = 0.7) to the corresponding 11, 21, 31, 45, and 2 hr post merozoite invasion time points of the microarray data set, despite the use of different methodologies (microarray vs RNA-seq) and the use of different *P. falciparum* strains (HB3 vs W2, respectively). Because of the higher sensitivity of RNA-seq, we were able to accommodate an additional 743 genes into the cascade-like transcriptome extending it to a total of 3110 genes (Figure 2B, Figure 2—source data 1). The remaining 495 genes in our RNA-seq data set lacked sufficient variation over the five time points for inclusion within the phaseogram. These genes, referred to as non-phasic genes, are nevertheless included in all analyses.

### Gene expression and translation are tightly coupled during the *P. falciparum* IDC

**Figure 3.**
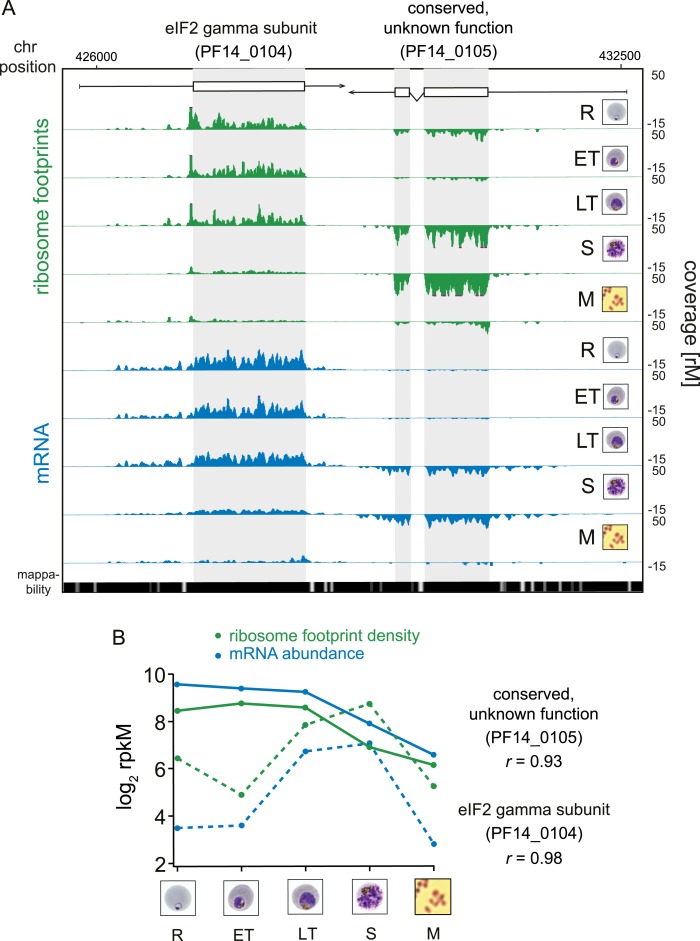
Transcription and translation are highly correlated. (**A**) Ribosome footprint (green) and mRNA (blue) coverage profiles of two neighbor genes, the eIF2 gamma subunit (PF14_0104) and the conserved protein PF14_0105 (CDS, white boxes; HMM-defined UTRs, black lines) in rings (R), early trophozoites (ET), late trophozoites (LT), schizonts (S), and merozoites (M). Mappability = mappability score at that position; range 0 (white) to 30 (black). rM = coverage (reads per million reads mapped). (**B**) mRNA and ribosome footprint density of the genes in (**A**) correlate during development. *r* = Pearson correlation coefficient between ribosome footprint density and mRNA abundance of each gene. **DOI:** http://dx.doi.org/10.7554/eLife.04106.010

While RNA-Seq reveals the abundance and architecture of individual mRNAs, ribosome profiling provides a complementary and quantitative measure of mRNA translation. Ribosome occupancy along the CDS results in a profile that indicates the timing and magnitude of translation of a given mRNA, thus quantitatively delineating regions of each mRNA molecule that are actually bound by 80S ribosomes (Ingolia et al., 2009). To inspect translation on a genome-wide scale, ribosome density values of each gene expressed in the data set were organized in the same order as the transcriptome. The translational profile of each gene displayed a cascade-like quality strikingly similar to the transcriptome (Figure 2B). Much like mRNA abundance, translation of phasic genes reaches a single maximum and a single minimum during the IDC. To determine the exact level of correlation between transcription and translation, we directly compared mRNA and ribosome footprint density measurements (Figure 2C). In general, translation is tightly correlated with transcription for all phasic and non-phasic genes in rings (*r* = 0.85), early trophozoites (*r* = 0.93), late trophozoites (*r* = 0.91), schizonts (*r* = 0.89), and purified merozoites (*r* = 0.86). This indicates that when an mRNA is detected in one stage it is associated proportionally with ribosomes within the same stage. An example pair of genes is shown in Figure 3A. Here, mRNA abundance profiles of eukaryotic translation initiation factor eIF2 gamma subunit (PF14_0104) and the conserved protein PF14_0105, show that peak mRNA abundance for these two genes occurs at two different stages, early and late, respectively. Examination of ribosome occupancy of both genes reveals a ribosome density accumulation profile within the coding sequence that mirrors their respective mRNA profiles. As for the majority of genes, ribosome footprint density and mRNA abundance for these two genes are highly correlated (*r* = 0.98 and 0.93 for PF14_0104 and PF14_0105, respectively), indicating that mRNA translation occurs proportionally during the same stages at which these genes are transcribed (Figure 3B; Supplementary file 1). Globally, 77% of genes expressed in at least three stages of the IDC display high Pearson correlation (*r* ≥ 0.7) between mRNA abundance and translation (Figure 3—figure supplement 1). Thus, our genome-wide analysis of translation establishes that for the majority of genes expressed during the IDC, transcription and translation occur proportionally.

**Figure 3—figure supplement 1.**
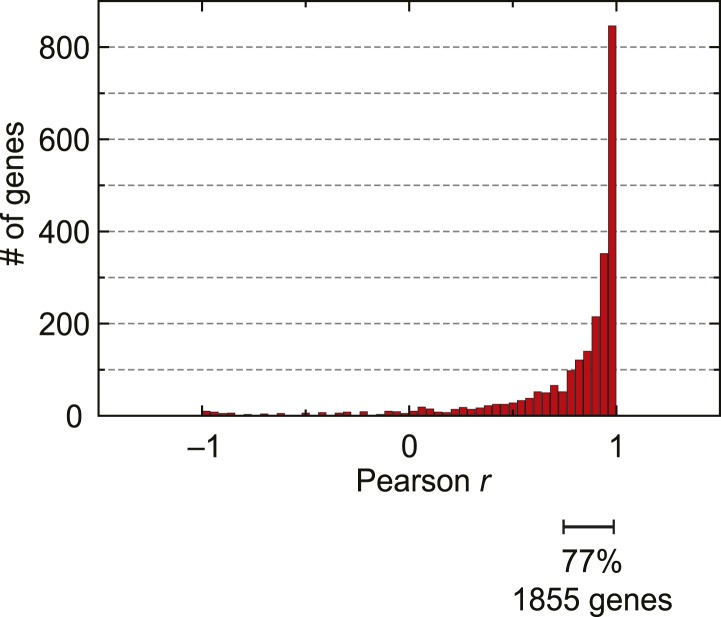
mRNA abundance and ribosome footprint density are highly correlated for the majority of genes expressed during the IDC. Pearson correlation of mRNA abundance and ribosome footprint density of every gene expressed in at least three stages (2412 genes). **DOI:** http://dx.doi.org/10.7554/eLife.04106.011

### Ribosome profiling reveals instances of translational control of gene expression

**Figure 4.**
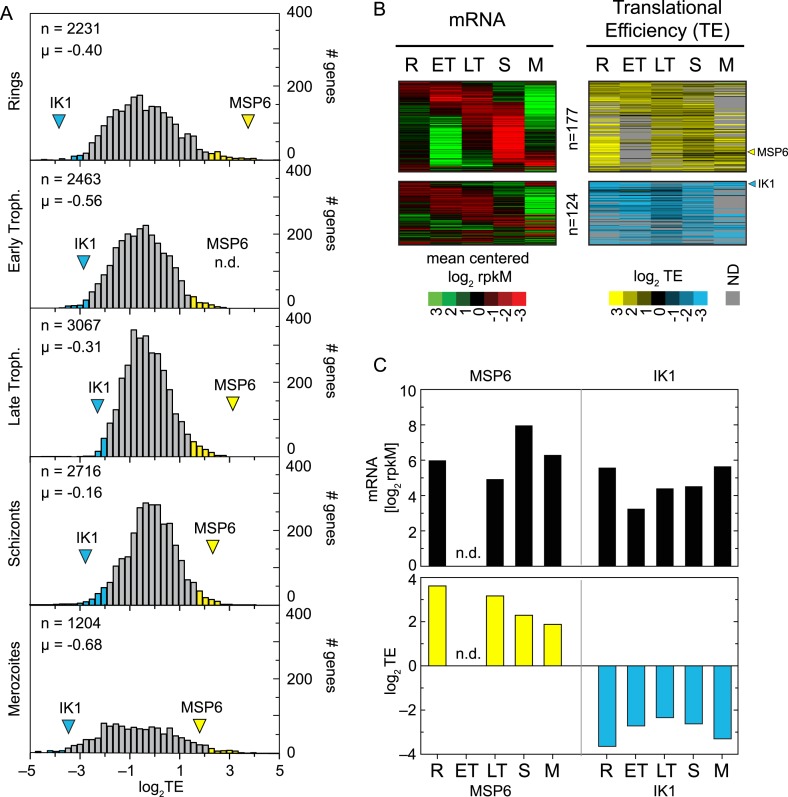
Genome-wide measurements of translation. (**A**) Translational efficiency distributions in each stage. Rings and merozoites have most extreme TE values; ± 2 SD above (yellow bars) and below (blue bars) the mean. TE values of translationally up-regulated merozoite surface protein (MSP6) and the eukaryotic initiation factor 2 alpha kinase 1 (IK1) (blue arrowhead) across the time course remain high and low, respectively. μ = mean log_2_TE, n = total number of genes. (**B**) mRNA abundance and translational efficiency heatmap of translationally up- and down-regulated genes (upper panel and lower panel, respectively). Note TE is independent of changes in mRNA abundance for all genes including MSP6 and IK1 (**C**). R = rings, ET = early trophozoites, LT = late trophozoites, S = schizonts, M = merozoites. n = number of genes, μ = mean, SD = standard deviation. **DOI:** http://dx.doi.org/10.7554/eLife.04106.012

Ribosome profiling allows the monitoring of translation rates through the simultaneous quantitative measure of mRNA abundance and ribosome density on mRNAs. The ratio of the footprint rpkM to the mRNA rpkM for any given gene represents its relative translational efficiency (TE) (Ingolia et al., 2009). To assess the dynamics of translational control and detect variations in control within and between developmental stages, we calculated the relative TE of all expressed genes in our data set (Figure 4A). The shape and the range of TE distributions obtained for each stage sampled is comparable to those seen in other eukaryotes (Ingolia et al., 2009; Dunn et al., 2013). Absolute mean translational efficiencies in all stages (log_2_TE µ_Rings_ = −0.43, log_2_TE µ_E.trophs._ = −0.56, log_2_TE µ_L.trophs_. = −0.31, log_2_TE µ_Schizonts_ = −0.16 and log_2_TE µ_Merozoites_ = −0.68) had a maximum difference of 1.47-fold observed between early trophozoites and schizonts. Translational efficiencies display a roughly 100-fold range in absolute values in each of the stages with the exception of the ring and merozoite stages, which exhibit more extreme values. In these stages, the distribution of absolute TE values displays an approximately fourfold larger spread than in early trophozoites, late trophozoites, or schizonts (Figure 4A, Figure 2—source data 1). In rings the gene with the largest TE is the merozoite surface protein 9 (PFL1385c, log_2_TE = 4.1) and the gene with the lowest TE is the FIKK family serine/threonine protein kinase (PF14_0734, log_2_TE = −5.1). In merozoites the largest and lowest TE values correspond to the serine repeat antigen 5 (SERA5, PFB0340c, log_2_TE = 4.0) and the alpha adenylyl cyclase (PF14_0788a-c, log_2_TE = −4.7), respectively.

To determine the contribution of translational efficiency to the dynamic range of gene expression, we examined the genes lying at the extremes of the TE distribution. For the purpose of this analysis, genes with a translational efficiency of two standard deviations above or below the mean in any of the stages were considered translationally up- or down-regulated, respectively. A total of 301 genes, 8.3% of the transcriptome, are translationally regulated by this metric, with 124 genes translationally down-regulated and 177 genes translationally up-regulated (Figure 4B, Figure 2—source data 1). The timing of maximum mRNA expression does not influence TE for either of these two groups. Translational efficiencies remain high for the translationally up-regulated and low for the translationally down-regulated genes in all the stages at which they are expressed, regardless of the stage of peak mRNA abundance, suggesting that translational efficiency is largely, but not completely, programmed by the mRNA sequence itself, rather than global factors. For example, translational efficiency of the merozoite surface protein 6 (MSP6, PF10_0346) remains high (log_2_TE ≥ 2) across all stages irrespective of variations in its mRNA abundance. In contrast TE values for the eukaryotic initiation factor 2alpha kinase 1 (IK1, PF14_0423) are among the lowest measured despite high mRNA abundance across all stages (Figure 4C).

**Figure 5.**
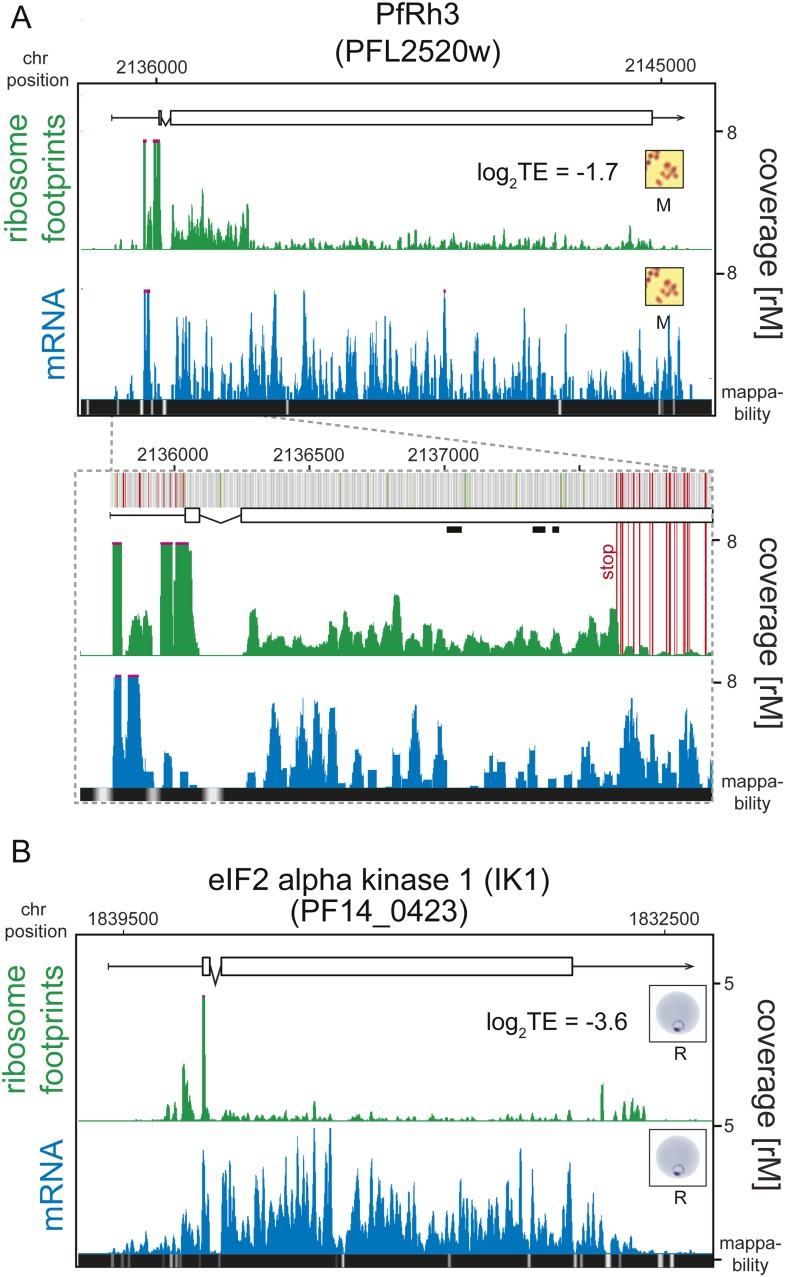
Translationally down-regulated genes have decreased CDS ribosome density. (**A**) Ribosome footprint (green) and mRNA (blue) profiles of the PfRh3 pseudogene (PFL2520w) in merozoites (M). In the detail the bars above the gene model indicate AUG, stop, and any other codon, in green, red, and gray, respectively. Boxes indicate the mapping location of peptides identified by mass spectrometry in gametocytes and sporozoites (Florens et al., 2002; Lasonder et al., 2002). Reduction of ribosome footprint coverage occurs upon encounter of consecutive stop codons (extended red lines). (**B**) eIF2α kinase (PF14_0423) gene in rings (R) showing ribosome footprint accumulation on the 5′ leader, 3′ UTR, and low translational efficiency of the CDS. (CDS, white boxes; HMM-defined UTRs, black lines. Mappability = mappability score at that position; range 0 (white) to 30 (black). rM = coverage (reads per million reads mapped). **DOI:** http://dx.doi.org/10.7554/eLife.04106.013

An examination of the 124 translationally down-regulated genes yielded some expected, and in some cases, unexpected findings. As would be expected, two pseudogenes, the ring-infected erythrocyte surface antigen 2 (RESA-2, PF11_0512) and reticulocyte binding protein homologue 3 (PfRh3, PFL2520w), represent a clear example of low translational efficiency. The PfRh3 pseudogene ribosome profile shows that translation of the 5′ end of this transcript occurs up until the encounter of several in-frame stop codons, causing the reduction in ribosome density from this point on (Figure 5A, Figure 5—figure supplement 1). This suggests that a truncated version of the PfRh3 protein is being produced in the W2 strain studied here. Evidence for peptides corresponding to the 5′ end of PfRh3 has been found in gametocytes and sporozoites (however not during the asexual stages) using mass spectrometry (Florens et al., 2002; Lasonder et al., 2002). We note that low levels of ribosomes can still be detected along the full length of this transcript in schizonts and merozoites. Whether these footprints derive from a low level of stop-codon read-through or accumulate via another unknown mechanism remains to be determined.

**Figure 5—figure supplement 1.**
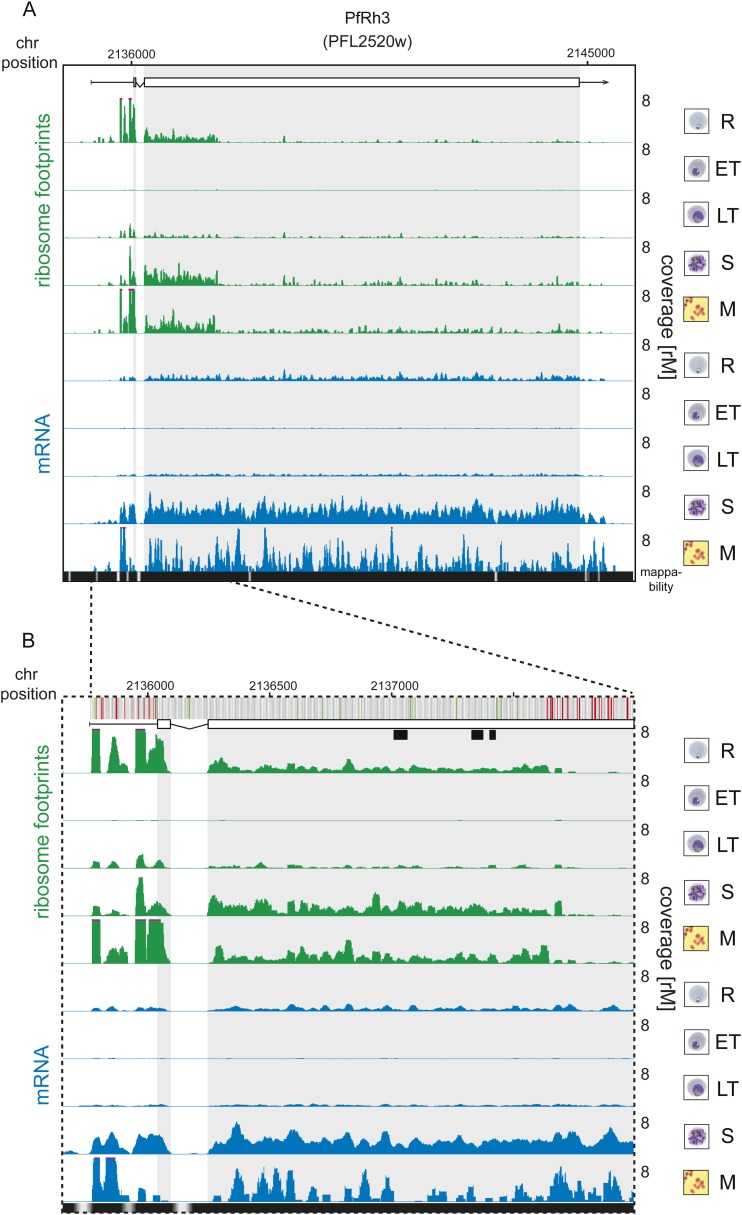
Translation of a truncated form of PfRh3 during the IDC. Ribosome footprint (green) and mRNA (blue) profiles of the PfRh3 pseudogene (PFL2520w) in rings (R), early trophozoites (ET), late trophozoites (LT), schizonts (S), and merozoites (M). (**A**) Translation of PfRh3 occurs until ribosomes dissociate upon the encounter of several consecutive in-frame stop codons (visible in **B**). (**B**) The vertical bars above the gene model indicate AUG, stop, and any other codon, in green, red, and gray, respectively. Boxes indicate the mapping location of peptides identified by mass spectrometry in gametocytes and sporozoites (Florens et al., 2002; Lasonder et al., 2002). **DOI:** http://dx.doi.org/10.7554/eLife.04106.014

**Figure 5—figure supplement 2.**
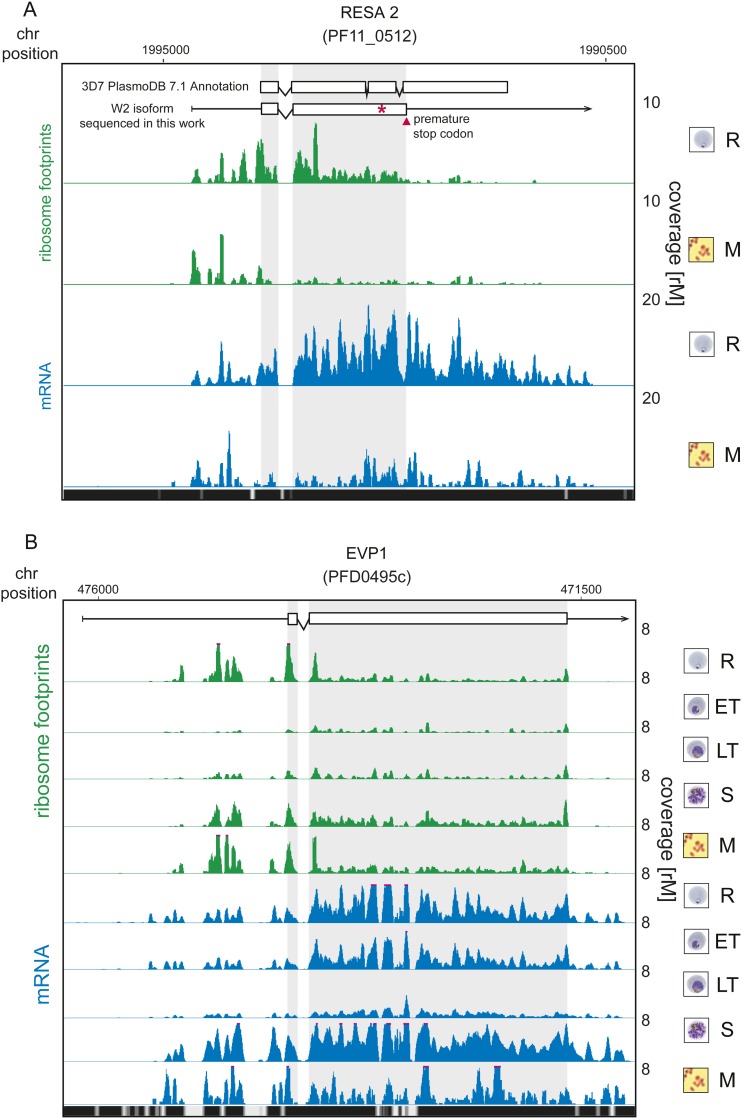
Translationally down-regulated genes have decreased CDS ribosome density. (**A**) Ribosome footprint (green) and mRNA (blue) profiles of the ring-infected erythrocyte surface antigen 2, RESA2 pseudogene (PF11_0512) in rings (R) and merozoites (M). Both, the annotated isoform from PlasmoDB version 7.1 and the gene model for the alternate isoform inferred using ribosome profiling and W2 genomic DNA sequencing data from this study is depicted (CDS, white boxes; HMM-defined UTRs, black lines). The red star indicates a homopolymeric tract in which a single base deletion causes a premature stop codon (red triangle), which coincides with the site of ribosome drop off. (**B**) Ribosome footprint and mRNA profiles of erythrocyte vesicle protein 1, EVP1 (PFD0495c). This gene is transcribed in all stages yet translational efficiencies are relatively low, as evidenced by a depletion of ribosomes on the CDS of the gene particularly in early trophozoites (log2TE = −2.6, −2.9, −1.0, −2.1, −1.4 in rings, early trophozoites, late trophozoites, schizonts, and merozoites, respectively). (CDS, white boxes; HMM-defined UTRs, black lines. Mappability = mappability score at that position; range 0 (white) to 30 (black). rM = coverage (reads per million reads mapped). **DOI:** http://dx.doi.org/10.7554/eLife.04106.015

Ring-infected erythrocyte surface antigen 2, RESA2 (PF11_0512) was first described as a pseudogene based on the presence of an internal stop codon (Cappai et al., 1992). Since then, transcription of this gene has been demonstrated both in vivo (Vazeux et al., 1993) and in vitro (Bozdech et al., 2003). RESA-2 is transcribed but poorly translated in rings, early trophozoites and merozoites (log_2_TE −3.2, −2.7, −2.9, respectively). Accordingly, the ribosome profile of this gene in merozoites shows a general depletion of ribosomes along the CDS (Figure 5—figure supplement 2). In rings, ribosome density diminishes at the second exon. To validate the RESA2 gene model, we used genomic DNA sequencing data derived from the *P. falciparum* W2 strain used in this study. We found that 69% (n = 151) of reads mapping to this locus support a single base deletion that creates a premature stop codon exactly at the site of ribosome footprint drop-off (Supplementary file 2). These data suggest that RESA2 is transcribed and actually translated into a shorter protein product of 461 amino acids. Whether or not the protein product is functional or undergoes post-translational degradation remains to be determined.

In addition to expected instances of translational regulation, our data permit the discovery of previously uncharacterized translational regulation, especially at the extremes of the TE distributions. One of the most notable examples of translational silencing is the eIF2α kinase IK1 (PF14_0423) for which ribosome footprints accumulate at the 5′ leader and 3′ UTR but not on the CDS, resulting in an extremely low translational efficiency (log_2_TE = −3.6) despite relatively high transcript abundance across all stages (Figure 5B). The mechanism by which this gene is maintained in a translationally down-regulated state is unknown. Another example is the erythrocyte vesicle protein 1 (EVP1, PFD0495c) for which abundant transcript levels can be detected across all stages, with peak mRNA abundance occurring in rings and schizonts (Figure 5—figure supplement 2). Protein levels, however, have been shown to be undetectable (Tamez et al., 2008). Here, we find that translational efficiencies of this gene were low across all stages and lowest in rings and early trophozoites (log_2_TE = −2.6 and −2.9, respectively) demonstrating that post-transcriptional regulation at the level of translation is, at least in part, responsible for its scarcity as a protein.

Thus, our ribosome profiling data set highlights instances of translational control of genes that may not be detected by proteomic methods. Indeed a search for mass spectrometric data showed no evidence for ∼70% of genes in this category (Aurrecoechea et al., 2009). Including the aforementioned examples, our data set describes a total 124 translationally down-regulated genes (listed in Figure 2—source data 1) for which translational efficiency values lie at the lower extremes of the distribution.

Protein products of translationally up-regulated genes are likely to be abundant and readily detected using mass spectrometry. Previous proteomic studies show protein evidence in the blood stages for almost all (171 of 177) well-translated genes identified here (Aurrecoechea et al., 2009; Pease et al., 2013). Mass spectrometric evidence for the remaining six genes is either absent (PFL0245w, PFL2510w, PF11_0204) or has only been found in sporozoites (PFE1615c, MAL7P1.300, PF13_0069a). Despite the lack of proteomic data, our data indicate that these genes are both transcribed and translated during the blood stages of the parasite. Whether post-translational control points exist for these proteins is unknown.

Among the top ten most highly translated genes are proteins involved in merozoite egress and invasion MSP3, 6, 7, and 9 (merozoite surface proteins PF10_0345, PF10_0346, PF13_0197, and PFL1385c), serine repeat antigen 5 (SERA5, PFB0340c), and RAP1, 2, and 3 (PF14_0102, PFE0080c, and PFE0075c, respectively) (Figure 2—source data 1). Interestingly, 73 (41%) of all translationally up-regulated genes can be assigned to the repertoire of canonical functions for merozoite egress and invasion described to date (Yeoh et al., 2007; Blackman, 2008; Hu et al., 2010; Farrow et al., 2011). Strikingly, for all genes in this set, maximum mRNA abundance is found during the late stages of the IDC (69 schizont and 4 merozoite stage mRNAs) yet for the majority (50, 70%) peak translational efficiency occurs in rings. Consistent with this, peptides for most of these merozoite function proteins (58 of 73) have been detected in rings (Oehring et al., 2012; Pease et al., 2013). This mode of translational regulation whereby late stage transcripts are highly translated in rings was not exclusively limited to genes related to merozoite egress and invasion. We found evidence for an additional 14 genes with this profile, including, aquaglyceroporin (PF11_0338, log_2_TE = 3.8), tubulin beta chain (PF10_0084, log_2_TE = 1.8), and early transcribed membrane protein 2 (PFB0120w, log_2_TE = 2.5).

Taken together these data demonstrate that transcription and translation are tightly correlated for the majority of genes expressed during the asexual life cycle of *P. falciparum* with few exceptions. These apply to a small subset of translationally down- and up-regulated genes for which translational efficiencies appear to be inherent properties of the mRNA, independent of changes in mRNA abundance. Genes in this category, especially those that exhibit high translational efficiencies, are enriched with functions associated with merozoite egress and invasion during the transition from late stages into rings.

### Ribosome occupancy of 5′ leaders is commonly found on genes expressed during the IDC

**Figure 6.**
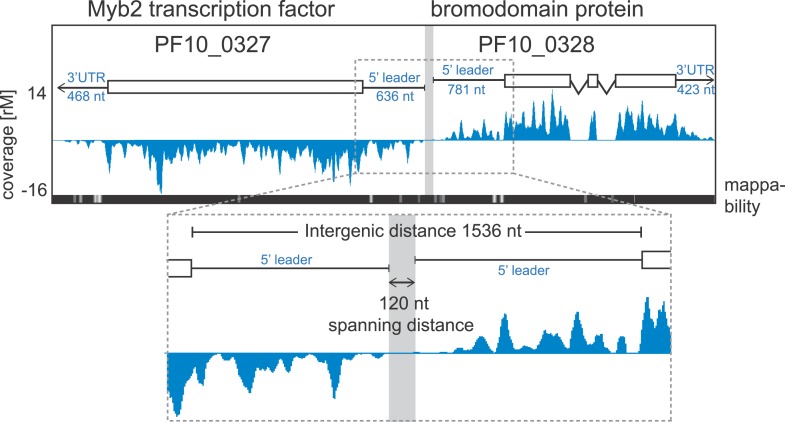
Example of extended transcript annotations using the HMM. 5′ leaders and 3′ UTRs of the gene pair Myb2 (PF10_0327) and bromodomain protein (PF10_0328) were defined using the HMM designed (see ‘Materials and methods’). The sizes of 5′ leaders and 3′ UTRs of these genes in the schizont stage are indicated. The intergenic region is 1536 nt and the spanning distance separating the 5′ leaders is 120 nt. Mappability = mappability score at that position; range 0 (white) to 30 (black). rM = coverage (reads per million reads mapped). **DOI:** http://dx.doi.org/10.7554/eLife.04106.016

Ribosome profiling provides position specific information along each transcript allowing the detection of changes in ribosome distribution on the mRNA and their relationship to translational efficiency. To look for ribosome occupancy features beyond the CDS of transcripts, we first took advantage of the deep coverage and strand specificity of our RNA-seq data to identify 5′ leaders and 3′ UTRs of the *P. falciparum* transcriptome. We constructed a hidden Markov model (HMM) to automatically delineate the boundaries of both 5′ leaders and 3′ UTRs for known gene models (see ‘Materials and methods’). Within the limits imposed by our data, we were able to describe 5′ mRNA leaders and/or 3′ UTRs for 3569 genes in at least one of the stages (Figure 6—figure supplement 1, Figure 2—source data 1). 5′ leaders are on average longer than 3′ UTRs in each of the stages and median lengths across stages vary to a larger degree for 5′ leaders (from 607 to 1040 nt) than for 3′ UTRs (518–622 nt). The longest 5′ mRNA leader was measured in late trophozoites (8229 nt) for the Ap2 transcription factor, PF11_0404, and the longest 3′ UTR stretched 4773 nt for 60S ribosomal protein L7-3, PF14_0231, in rings. An example pair of genes with mapped 5′ leaders and 3′ UTRs is shown in Figure 6. Here, our HMM predicts a 636 nt and a 781 nt 5′ leader and a 468 nt and 423 nt 3′ UTR for the Myb2 transcription factor (PF10_0327) and the bromodomain protein (PF10_0328), respectively. These genes, encoded on opposite strands, share a 1536-nt intergenic sequence; however, the span between the region delimited by their 5′ leader sequence is only 120 nt and presumably harbors their respective promoters.

**Figure 6—figure supplement 1.**
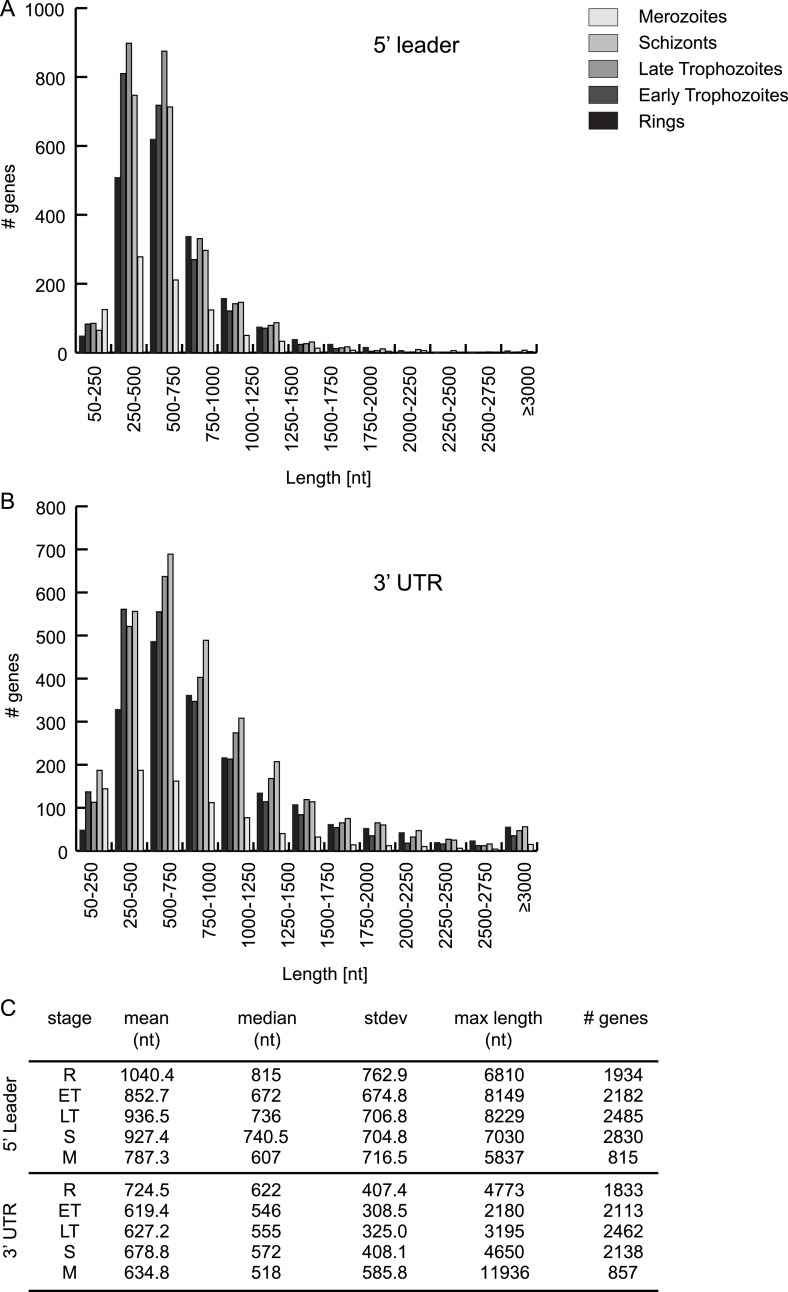
HMM-defined 5′ leader and 3′ UTR characteristics. 5′ leader (**A**) and 3′ UTR (**B**) length distribution and their statistics (**C**) per stage. **DOI:** http://dx.doi.org/10.7554/eLife.04106.017

**Figure 7.**
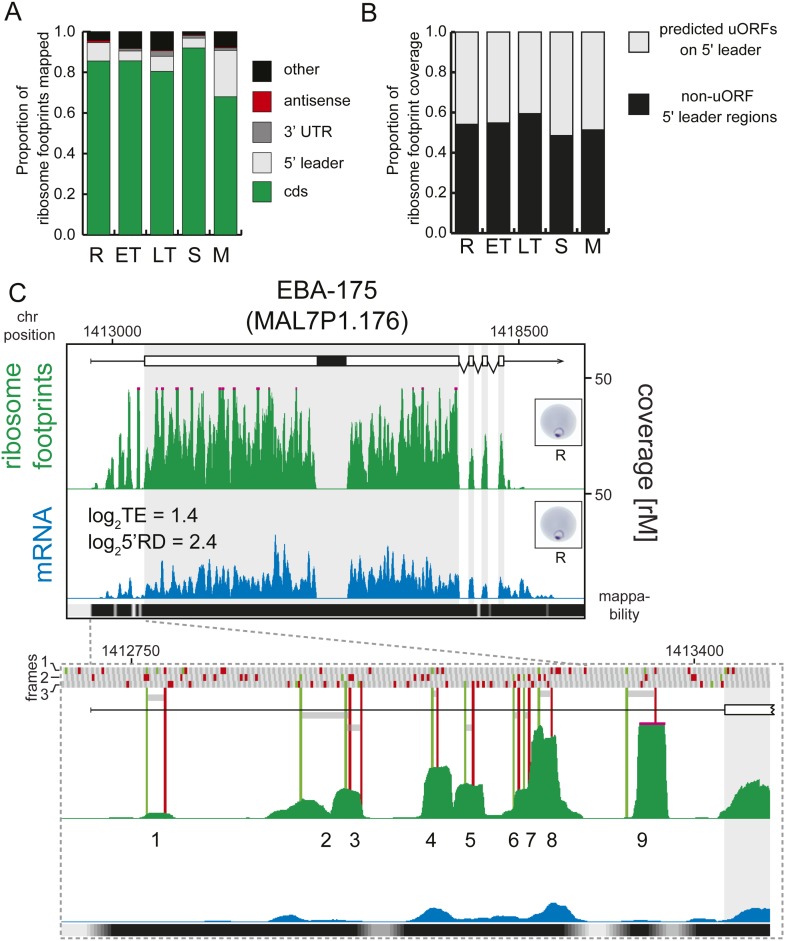
Transcripts accumulate ribosome density within the 5′ leader. (**A**) Proportion of mRNA or ribosome footprint reads mapping to CDS, to HMM-defined 5′ leaders and 3′ UTRs, antisense to annotated coding genes or to other regions of the genome such as mitochondria, plastid, tRNA, rRNA, ncRNA, and 5′ leader and 3′ UTR regions not defined by the HMM. (**B**) Proportion of ribosome footprints mapping inside or outside predicted uORFs in the HMM-defined 5′ leaders. (**C**) Ribosome footprint (green) and mRNA (blue) profiles of the EBA-175 (MAL7P1.176) gene in rings (R) showing ribosome footprint accumulation on the 5′ leader. In the detail, the bars above the gene model indicate AUG, stop, and any other codon, in green, red, and gray, respectively and in all three possible frames. Gray bars indicate the 9 uORFs present in the 5′ leader, starting with an AUG (green line) and ending with a stop codon (red line). Black bar inside CDS indicates a deletion specific to the W2 strain used in this study. CDS, white boxes; HMM-defined UTRs, black lines. Mappability = mappability score at that position; range 0 (white) to 30 (black). rM = coverage (reads per million reads mapped). **DOI:** http://dx.doi.org/10.7554/eLife.04106.018 10.7554/eLife.04106.019Figure 7—source data 1.Predicted uORFs.**DOI:** http://dx.doi.org/10.7554/eLife.04106.019

Next, using mRNA boundaries derived from our data, we analyzed ribosome distribution along each transcript during life cycle progression. More than 80% of the ribosome footprints in rings, early trophozoites, late trophozoites, and schizonts, were mapped to CDS regions of the genome, except in merozoites, where only 68% were mapped to the CDS (Figure 7A). On average less than 1% of all reads obtained were mapped to 3′ UTRs in each stage, and most transcripts had no observed footprints past the stop codon. In contrast, footprints were far more common in 5′ leaders (9.1%, 4.8%, 7.5%, and 4.8% in rings, early trophozoites, late trophozoites, and schizonts, respectively) particularly in merozoites (23%). Footprint enrichment is specific to 5′ leaders and not due to non-specific background since this would result in an increase of footprints mapping evenly along the length of the transcript, including the 3′ UTR, and not just the 5′ leader. Furthermore, these footprints most likely represent ribosomes because they derive from the 80S monosome fraction of the sucrose gradient, and their footprint read length distributions are equal to those of CDS mapping footprints, whereas they are significantly divergent from rRNA or tRNA read length distributions (Figure 7—figure supplement 1).

**Figure 7—figure supplement 1.**
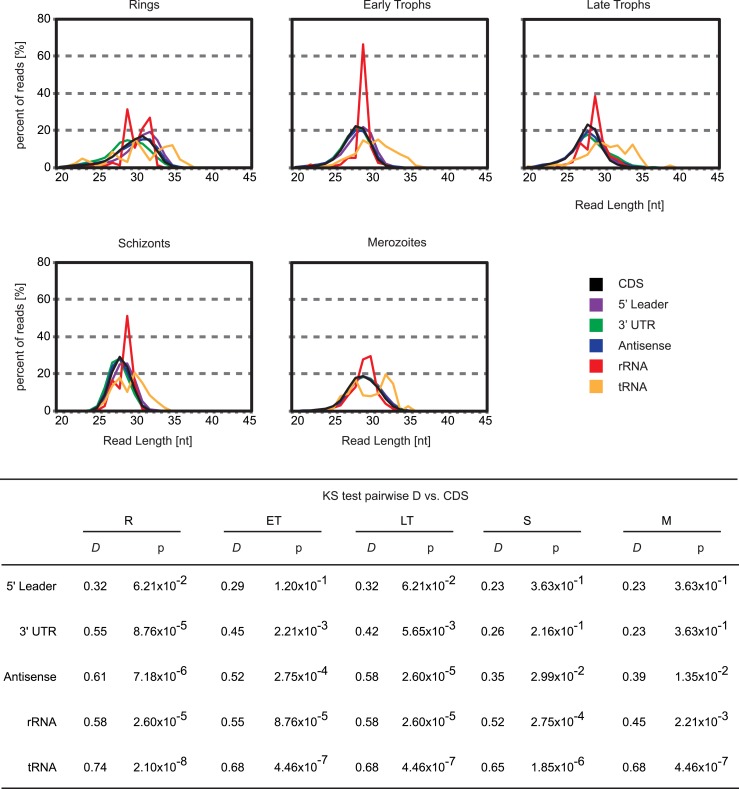
5′ leader footprints are derived from ribosomes. Ribosome footprint read length distributions for reads mapping either to CDSs, 5′ leaders, 3′ UTRs, antisense, rRNAs or tRNAs are plotted. Read lengths of rRNA and tRNA mapping footprints are significantly different than those mapping the 5′ leader, the CDS, or the 3′ UTR of transcripts in all stages. KS = Kolmogorov–Smirnov test. D = KS test statistic. R = rings, ET = early trophozoites, LT = late trophozoites, S = schizonts, M = merozoites. **DOI:** http://dx.doi.org/10.7554/eLife.04106.020

**Figure 7—figure supplement 2.**
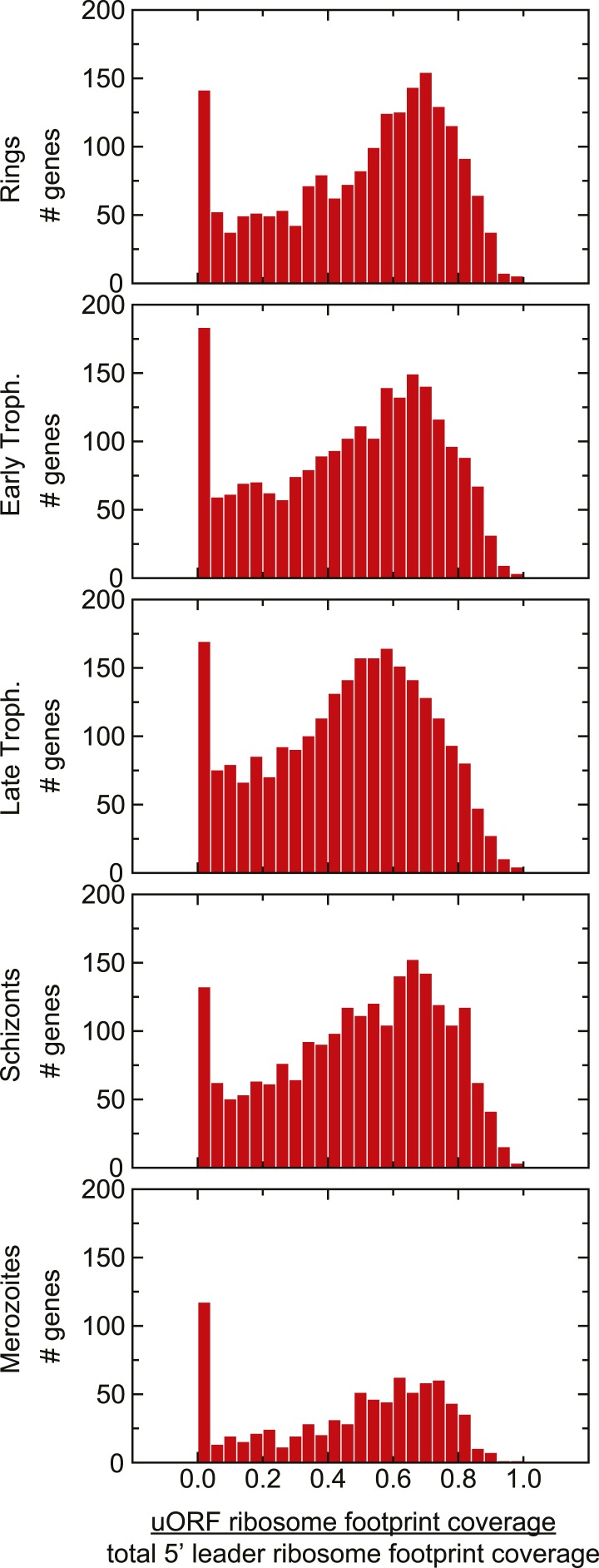
Distribution of uORF coverage on 5′ leaders of genes expressed during the IDC. The proportion of ribosome footprints mapping inside predicted uORFs was calculated for each gene expressed in each stage. The median of each of these distributions is ∼0.5. **DOI:** http://dx.doi.org/10.7554/eLife.04106.021

**Figure 7—figure supplement 3.**
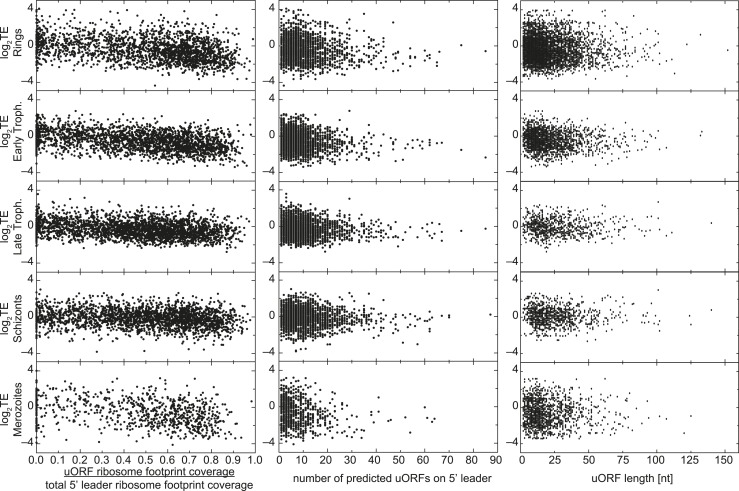
uORFs present on 5′ leaders have no effect on TE. Translational efficiency (log2TE) for all genes expressed in each stage is plotted against the proportion of reads mapping within uORFs, the number of predicted uORFs, or the length of predicted uORFs in the 5′ leader. No direct relationship between these parameters can be observed. **DOI:** http://dx.doi.org/10.7554/eLife.04106.022

**Figure 7—figure supplement 4.**
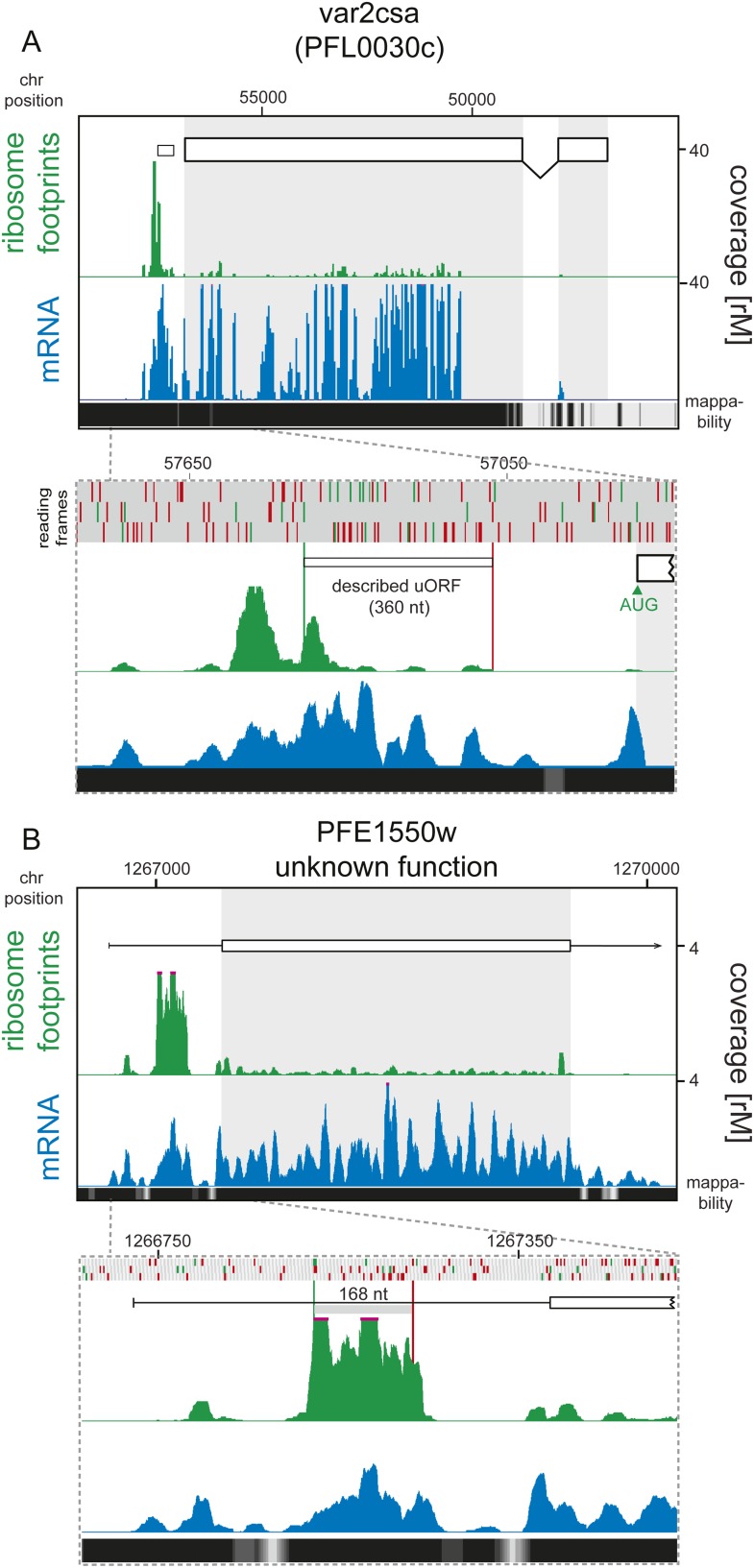
Detection of ribosome density on uORFs. (**A**) Ring stage mRNA (blue) and ribosome footprint (green) profiles of VAR2CSA (PFL0030c) are shown. There is virtually no ribosome density on transcript CDS (log2TERings = −4.2). Ribosomes do accumulate on the previously described (Amulic et al., 2009) 360 nt uORF (white box). This region is depicted in more detail in the panel below where the amino acids, AUGs and stop codons of each of the three reading frames are denoted with gray, green, and red bars, respectively. Note that ribosomes start accumulating upstream of the previously described uORF. Mappability at the 3′ end of this antigenic variation gene is poor and therefore no mRNA read coverage can be detected here. (**B**) Ring stage mRNA (blue) and ribosome footprint (green) profiles of PFE1550w (unknown function) are shown. Translational efficiency of the CDS is log2TERings = −3.6 in rings. 90% of ribosome footprints that map to the 5′ leader of this gene accumulate on one of the six predicted uORFs (detailed figure below). The predicted uORF is 168 nt (56 aa). Mappability = mappability score at that position; range 0 (white) to 30 (black). rM = coverage (reads per million reads mapped). **DOI:** http://dx.doi.org/10.7554/eLife.04106.023

**Figure 7—figure supplement 5.**
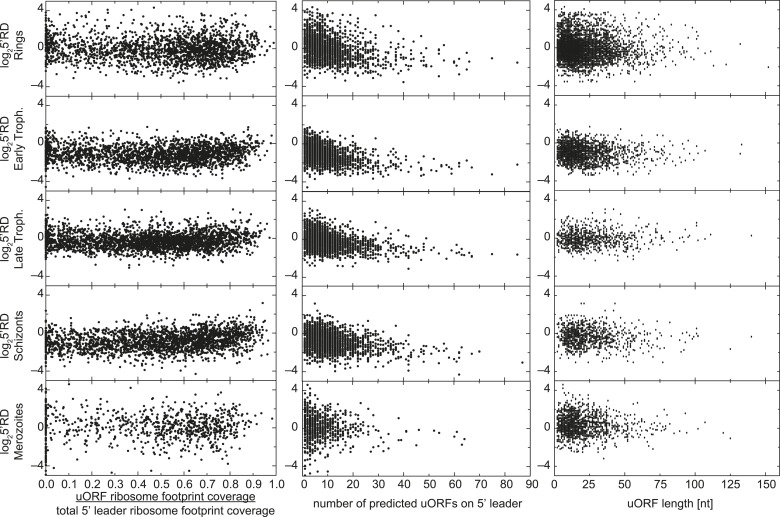
uORFs present on 5′ leaders have no effect on TE. Ribosome density on the 5′ leader (log25′RD) for all genes expressed in each stage is plotted against the proportion of reads mapping within uORFs, the number of predicted uORFs, or the length of predicted uORFs in the 5′ leader. No direct relationship between these parameters can be observed. **DOI:** http://dx.doi.org/10.7554/eLife.04106.024

**Figure 7—figure supplement 6.**
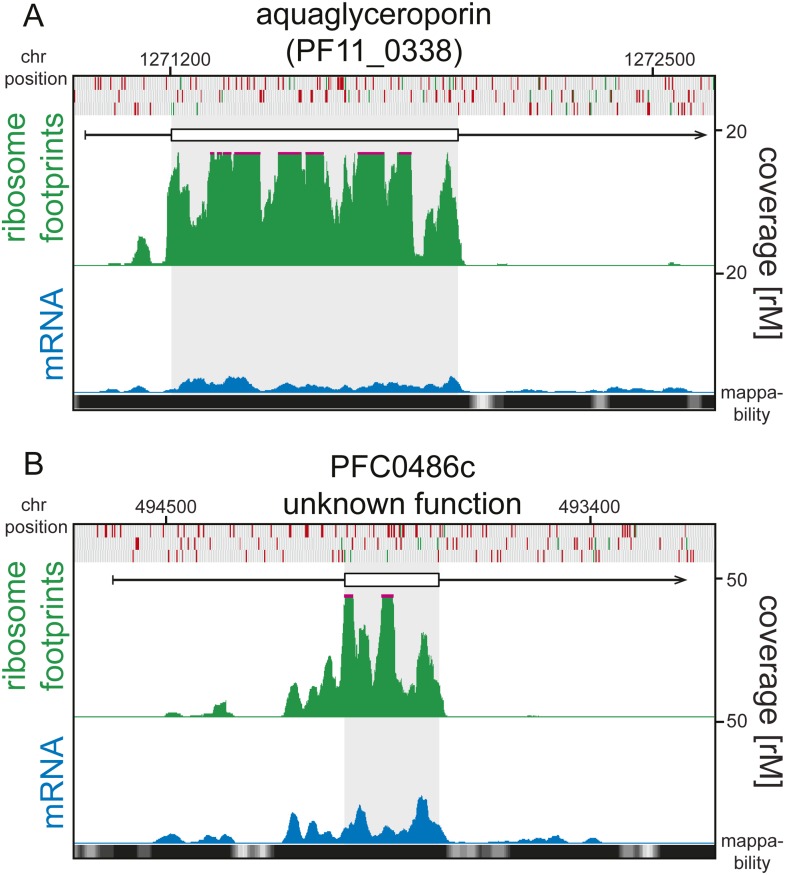
5′ ribosome density can be found on 5′ leaders devoid of AUGs. Ring stage mRNA (blue) and ribosome footprint (green) profiles of (**A**) aquaglyceroporin (PF11_0338) and (**B**) PFC0486c (unknown function) are shown. Both genes display high ribosome density on their 5’ leaders and these are devoid of AUGs. Mappability = mappability score at that position; range 0 (white) to 30 (black). rM = coverage (reads per million reads mapped). **DOI:** http://dx.doi.org/10.7554/eLife.04106.025

Upstream open reading frames are a major source for 5′ leader ribosome density found from yeast to humans (Ingolia et al., 2009; Brar et al., 2012), and these have been shown to play a role in translational regulation of the downstream ORF in a few well-studied examples (Morris and Geballe, 2000). In *P. falciparum*, ribosomes have been suggested to accumulate on 5′ leaders of genes displaying a delay in translation presumably due to long uORFs (Bunnik et al., 2013).

We defined 36,086 possible uORF regions in the 5′ leaders of genes expressed during the *P. falciparum* IDC using a liberal definition that includes any stretch of at least two amino acids, starting with an AUG codon (Figure 7—source data 1). Regardless of stage, half of the total ribosome footprint coverage in 5′ leaders, in aggregate, or on a gene-by-gene basis did not overlap with these predicted uORFs (Figure 7B, Figure 7—figure supplement 2). We could find no significant correlation between the number of uORFs per gene, the uORF lengths, or the degree to which ribosome density was enriched in uORFs with translational efficiency (Figure 7—figure supplement 3). For example, erythrocyte binding antigen-175 (EBA-175, MAL7P1.176) is well translated in rings (log_2_TE = 1.4) and displays a large amount of 5′ leader ribosome occupancy. Half (49%) of the reads map within the nine predicted uORFs on the 5′ leader of this gene, the other half maps outside these uORFs (Figure 7C). Using this liberal definition of an uORF, the data do not support an association between ribosome occupancy in these regions, nor does it support an association between the presence of these regions and translational efficiency.

Nonetheless, there exist at least two clear exceptions. First, we were able to validate translation of the reported uORF present in the 5′ leader sequence of the var2csa mRNA which is expressed only in rings (Amulic et al., 2009). The majority of ribosome footprint density localizes to this uORF, and to a second one just upstream, while the var2csa coding sequence is largely devoid of footprints (log_2_TE_Rings_ = −4.2, Figure 7—figure supplement 4), consistent with its translational repression during growth in the absence of plancental tissue. Second, another striking example of uORF translation was found on PFE1550w (unknown function) for which the ratio of uORF to total 5′ leader mapping reads is 0.9 (Figure 7—figure supplement 4). Indeed, ribosome footprint density is concentrated on one of the 6 uORFs predicted in the 5′ leader of this gene, 56 amino acids long. This gene is also translationally down-regulated in all stages (log_2_TE = −2.7 on average). These two genes represent exceptional cases for which uORF translation negatively correlates with translation of the downstream ORF.

Aside from these two exceptions, for the vast majority of genes, ribosome occupancy appears spread along 5′ leaders and not preferentially concentrated within possible uORFs. For this reason, we calculated 5′ leader ribosome density (5′RD) for each gene expressed during the IDC, defined as upstream ribosome occupancy normalized for mRNA expression level and size of the leader sequence (5′ leader ribosome footprint rpkM/5′ leader mRNA rpkM) (Figure 2—source data 1). No positive correlation exists between the number of uORFs per gene, the uORF lengths, or the degree to which ribosome density is enriched in uORFs and 5′RD, reinforcing the notion that uORFs are not a requisite for ribosome association to 5′ leaders (Figure 7—figure supplement 5). In fact 5′ ribosome density can be found on transcripts with 5′ leaders completely devoid of AUGs, and thus, without uORFs by definition, such as the highly translated aquaglyceroporin (log_2_TE = 3.8 and log_2_5’RD = 2.9 in rings), and PFC0486c (unknown function, log_2_TE = 1.6 and log_2_5′RD = 1.1 in rings) (Figure 7—figure supplement 6).

**Figure 8.**
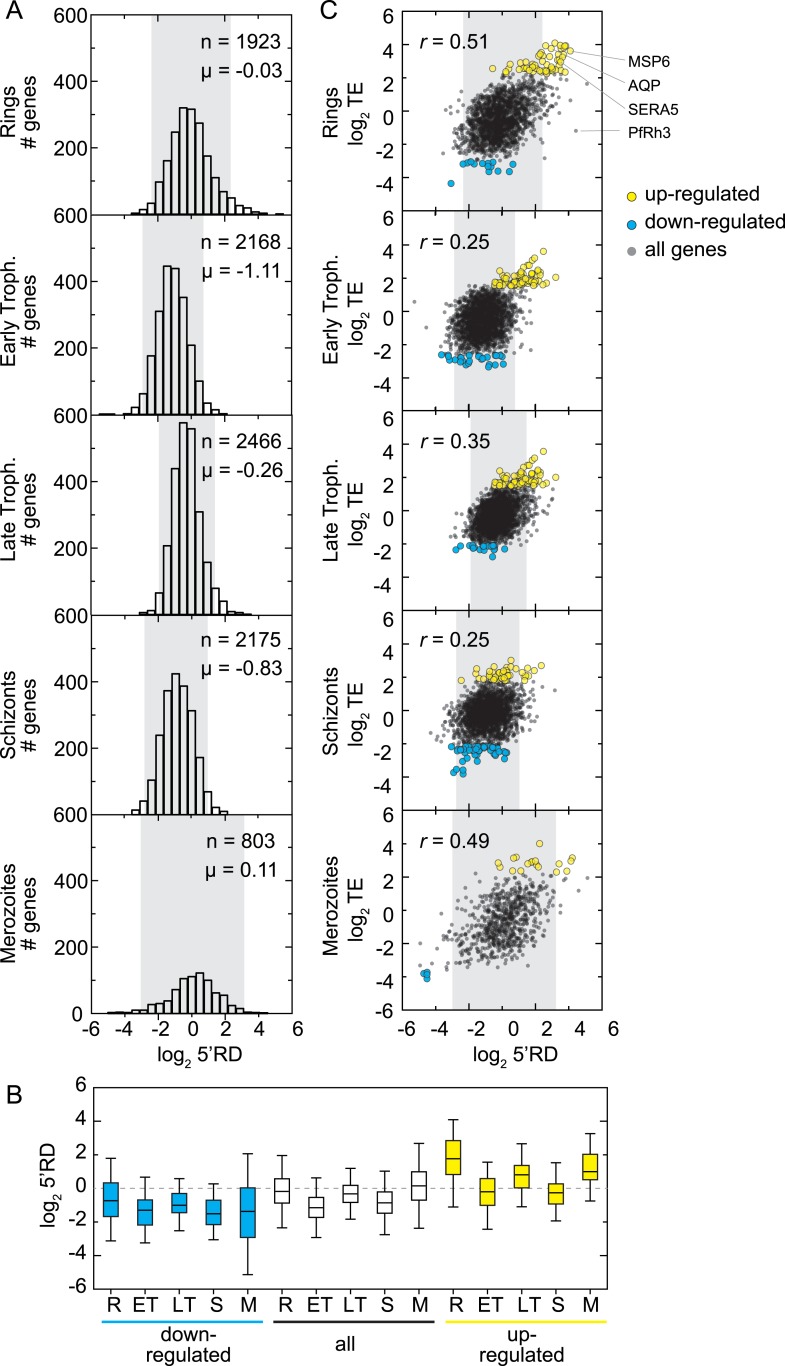
5′ ribosome density is commonly found on genes expressed during the IDC. (**A**) 5′ RD distributions in each stage. Transcripts in rings and merozoites have on average higher 5′ RD values; ± 2 SD values lie outside gray shade. μ = mean log_2_5′RD, n = total number of genes. (**B**) 5′RD values of the translationally up-regulated set of genes (yellow boxes) are relatively higher (average log_2_5′RD R = 1.73, ET = −0.26, LT = 0.78, S = 0.30, M = 1.16.) than the rest (white boxes) or the set of down-regulated (blue buxes) genes. (**C**) 5′RD weakly correlates with translational efficiency. The translationally up-regulated gene set (yellow circles) is associated with high 5′RD, particularly in rings. The translationally up-regulated genes merozoite surface protein (MSP6), aquaglyceroporin (AQP), serine repeat antigen (SERA5), and the reticulocyte binding protein homologue 3 (PfRh3) are pointed out. *r* = Pearson correlation coefficient. **DOI:** http://dx.doi.org/10.7554/eLife.04106.026

Overall, rings and merozoite stage parasites were found to express transcripts with the highest 5′RD (mean log_2_5′RD −0.03, and 0.11, respectively) relative to early trophozoites, late trophozoites, and schizonts (mean log_2_5′RD −1.11, −0.26, −0.83), where the range of 5′RD values is also narrower (Figure 8A). Interestingly, among genes at the extremes of the 5′RD distributions (mean ± 1 SD), we also found many of our identified translationally up- and down-regulated transcripts (66% and 40%, respectively). On average, 5′RD was enriched on translationally up-regulated transcripts (mean log_2_5′RD = 0.83) and depleted for translationally down-regulated transcripts in all stages (mean log_2_5′RD = −1.11), suggesting the possibility that 5′RD is a byproduct of translational efficiency itself (Figure 8B).

In order to determine whether a direct relationship between 5′RD and translational efficiency of the downstream ORF exists, we compared these values for each gene. 5′RD positively correlates, albeit moderately, with translational efficiency in all stages, particularly in rings and merozoites (*r* = 0.51 and 0.49, respectively). We focused on the subset of genes with highest and lowest 5′RD values (mean ± 2 SD) and found that only a fraction of the translationally up- and down-regulated genes overlap with this category of extreme 5′RDs in each stage (Figure 8C). The largest overlap occurred in rings where the highest 5′RD values were found in 43% (31 genes) of the translationally up-regulated genes, including MSP6, AQP, and SERA5. These results indicate that while in general a correspondence between 5′RD and translational efficiency exists, one is not necessarily predictive of the other and exceptions apply. This is the case, for example, of the translationally down-regulated transcript of the pseudogene PfRh3, which in rings has the second highest 5′RD value (log_2_5′RD = 5.1).

In summary, our data establish ribosome accumulation on 5′ leaders as a common feature of transcripts expressed during the IDC. Ribosome density is not restricted to predicted uORFs present within these regions and, with few exceptions, the uORF number, length, or coverage level, is not a requirement for 5′ ribosome density and has no measurable effects on the translation of the downstream ORF. Even though 5′RD is more commonly found on 5′ leaders of highly translated transcripts, this is not a universal trend since only a moderate correlation exists between 5′RD and the translational efficiency of the downstream ORF.

### 3′ UTR ribosome occupancy is rare

**Figure 9.**
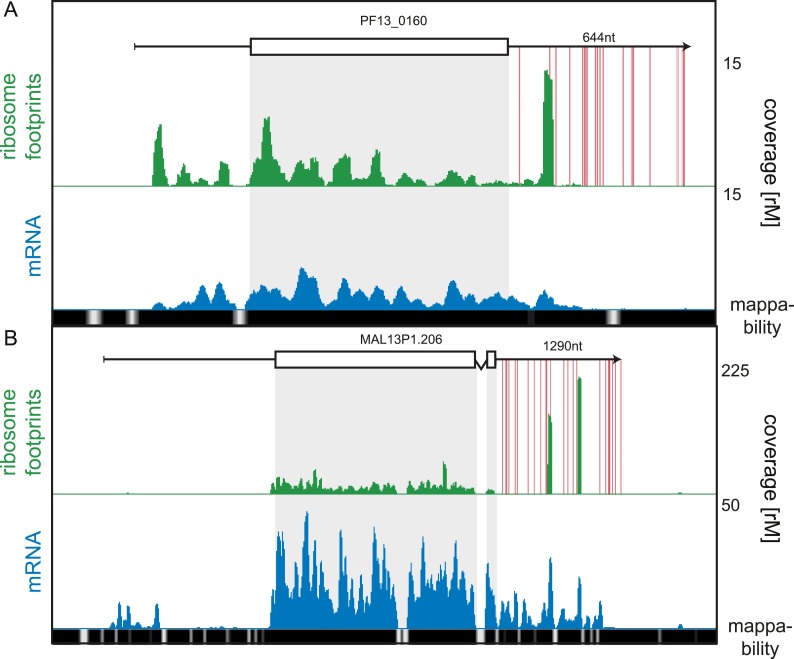
3′ UTR ribosome density. (**A**) Late trophozoite stage mRNA (blue) and ribosome footprint (green) profiles of the conserved plasmodium protein, PF13_0160. Ribosomes can be detected up to ∼130 nt beyond the stop codon on the 3′ UTR and accumulate in a single large peak. Red lines indicate in-frame stop codons on the 3′ UTR. (**B**) Two large peaks of ribosome footprint density can be detected 560 nt and 860 nt downstream from the stop codon in the 3′ UTR of the sodium-dependent phosphate transporter, MAL13P1.206. **DOI:** http://dx.doi.org/10.7554/eLife.04106.027 10.7554/eLife.04106.028Figure 9—source data 1.Genes with 3′ UTR ribosome occupancy.**DOI:** http://dx.doi.org/10.7554/eLife.04106.028

While our data showed 3′ UTRs to be relatively depleted of ribosomes, we searched for rare cases of high 3′ UTR ribosome density, possibly arising from stop codon read-through, alternative stop codon usage, or re-initiation of downstream ORFs (Dunn et al., 2013; Guydosh and Green, 2014). We systematically searched for transcripts for which coverage, in a sliding window of 30 nt, was greater in the 3′ UTR than the CDS, and found 19 genes meeting this criterion. These genes could be qualitatively divided into two categories: 14 with putative stop codon read-through and/or alternate stop codon usage and 5 genes for which the origin of the 3′ UTR density is unclear (listed in Figure 9—source data 1). An example of stop codon read-through is the conserved plasmodium protein (PF13_0160), shown in Figure 9A. Ribosomes not only extend beyond the annotated stop codon of this transcript but also skip subsequent in-frame stop codons present on the predicted 644 nt 3′ UTR. Interestingly, ribosome footprints accumulate in a single large peak approximately 130 nt downstream of the annotated stop codon. On the 1290 nt 3′ UTR of the sodium-dependent phosphate transporter (MAL13P1.206), two large peaks of ribosome footprint density, one approximately 560 nt and the other 860 nt from the stop codon, can be observed (Figure 9B). The origin of these footprints is unclear, and it is possible that these are the product of nuclease protection by RNA-binding proteins that co-sediment with the 80S monosome. To confirm that 3′ UTR mapping reads are derived from ribosome footprints, we compared their cumulative read length distributions against a typical CDS footprint read length distribution (Figure 9—figure supplement 1). For the 16 of the 19 genes we observed no significant difference in footprint size distributions localized to the CDS compared with the 3′ UTR. For the remaining three genes, the sodium-dependent phosphate transporter (MAL13P1.206), the acyl-Coa synthetase (PFD0085c), and the conserved plasmodium protein (PF13_0160), 3′ UTR footprint size distributions were divergent from those on the CDS, implying that footprints found on these genes' 3′ UTRs may be produced by nuclease protection of these regions by factors other than ribosomes that co-sediment with 80S ribosomes.

**Figure 9—figure supplement 1.**
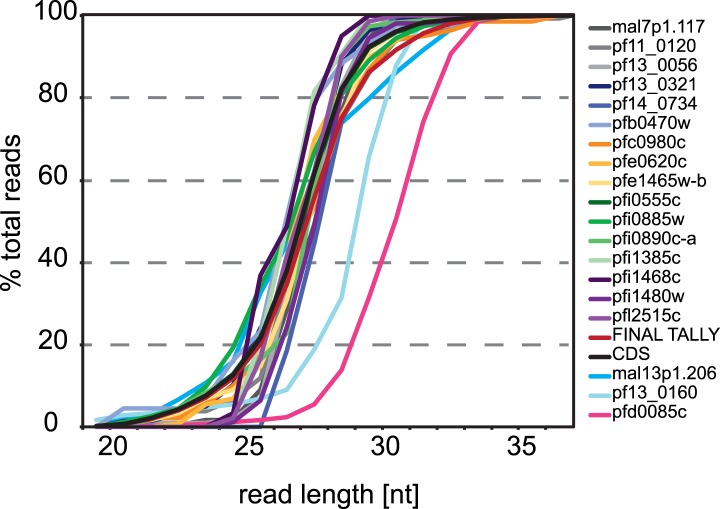
3′ UTR ribosome footprint size distribution. Cummulative read length distributions of all reads mapping to the 3′ UTR of the 19 genes with 3′ ribosome density identified compared to the read length distributions of reads mapping to all CDSs in the late trophozoite stage (black line). Footprint length distributions for MAL13P1.206, PF13_0160, and PFD0085c are least similar to the ribosome footprints that map to the CDS. **DOI:** http://dx.doi.org/10.7554/eLife.04106.029

### Antisense detection

Antisense transcription plays an important role in gene regulation from bacteria to humans, and while its role is increasingly studied in these organisms (Faghihi and Wahlestedt, 2009), less is known about its relevance in *P. falciparum*. Previous serial analysis of gene expression (SAGE) (Patankar et al., 2001), nuclear run-on experiments (Militello et al., 2005), and more recently antisense splicing events detected by RNA-seq (López-Barragán et al., 2011; Sorber et al., 2011), suggest that antisense RNAs are synthesized by RNA pol II and may constitute up to ∼12% of the erythrocytic-stage steady-state RNA (Gunasekera et al., 2004), yet their presence and biological role, if any, remains unclear. A more recent study found no correlation between natural antisense transcript levels and protein abundance (Siegel et al., 2014).

The 30 nt fragmentation and RNA-ligase-based library preparation method employed here affords exquisite strand specificity by minimizing artifacts associated with random priming during reverse transcription. As evidence of this specificity, the highest expressed gene in our data set, histone h2a (PFF0860c), yielded a total of 765,510 reads on the sense strand, and only two reads on the antisense strand, corresponding to a sense:antisense ratio greater than 10^5^. Furthermore, our HMM mapping of 5′ leaders and 3′ UTRs facilitates the differentiation between independently transcribed antisense RNA and transcripts that occur by virtue of being part of an adjacent gene. We took advantage of the nature of our data set to identify antisense transcripts and looked for effects on sense mRNA translation.

**Figure 10.**
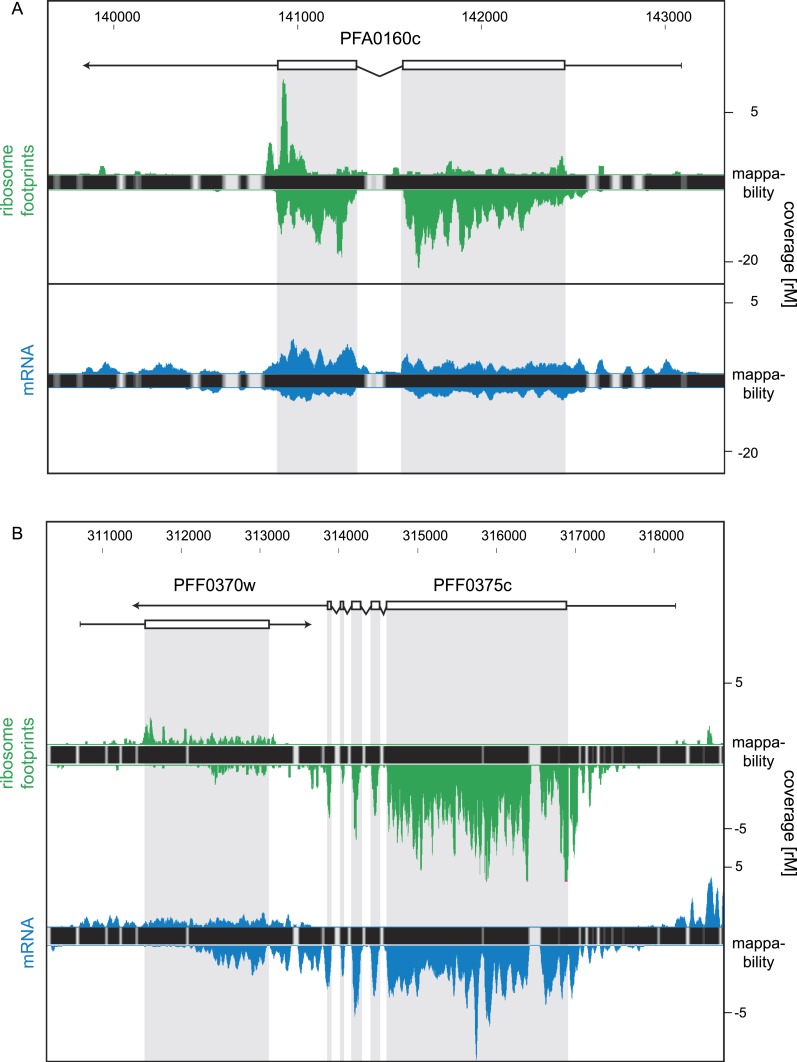
Strand-specific libraries can distinguish antisense from sense gene transcription. (**A**) Schizont stage mRNA (blue) and ribosome footprint (green) profiles of the nucleoside transporter pfNT4 (PFA0160c). The antisense transcript covers the full extent of the sense transcript and displays ribosome density. (**B**) An example of antisense reads originating from a neighboring UTR in the schizont stage. The antisense reads in the para-hydroxybenzoate polyprenyltransferase (PFF0370w) stem from the 3′ UTR of the neighboring conserved plasmodium protein (PFF0375c). **DOI:** http://dx.doi.org/10.7554/eLife.04106.030 10.7554/eLife.04106.031Figure 10—source data 1.Antisense transcripts.**DOI:** http://dx.doi.org/10.7554/eLife.04106.031

For this analysis only, we relaxed our stringent coverage threshold from ≥32 rM to ≥16 rM for inclusion of antisense transcripts. We based our threshold on the presence of an antisense transcript to the sexual stage specific gene pfs16 (PFD0310w) confirmed by strand-specific RT-PCR (Figure 10—figure supplement 1). This antisense is predicted by the HMM to be ∼4 kb, extending over the complete coding sequence and beyond and with a coverage level of 23 rM over the sense CDS. Using the 16 rM threshold, we detected 84 antisense transcripts to several known ORFs (listed in Figure 10—source data 1), including the nucleoside transporter pfNT4 (PFA0160c) depicted in Figure 10A. The merozoite stage contained the highest number of antisense transcripts (46), and the fewest (13) were found in early trophozoites. Manual inspection revealed that in 63% of these instances, the putative antisense transcript actually emanates from the 5′ leader or 3′ UTR of a neighboring gene (not defined by the HMM). Antisense reads for the para-hydroxybenzoate polyprenyltransferase (PFF0370w), for example, are actually derived from the 3′ UTR of the neighboring conserved protein PFF0375c (Figure 10B).

**Figure 10—figure supplement 1.**
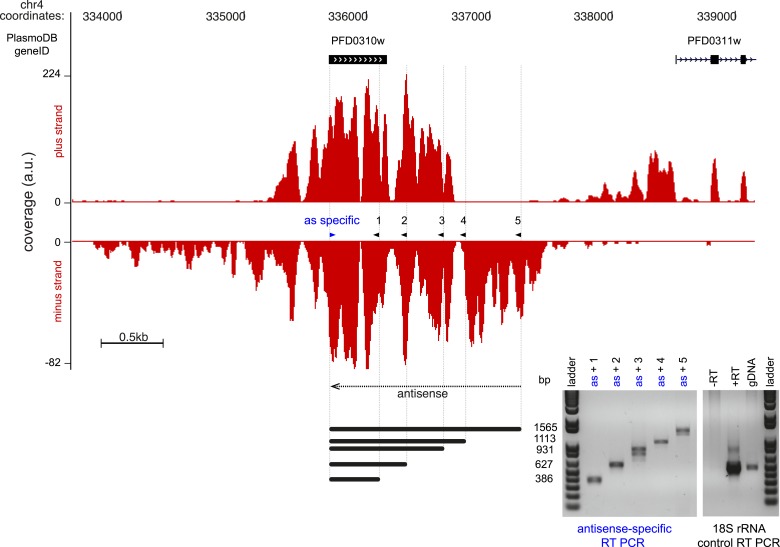
Strand-specific RT-PCR detection of the antisense to Pfs16. Read coverage on the plus and minus strand of the stage-specific protein precursor Pfs16 (PFD0310w) locus. The gene is encoded on the plus strand and the antisense transcript covers and extends beyond the sense transcript ∼3.7 kb. The strand-specific primer was used for both reversetranscription and as forward primer for the PCR (blue arrowhead). The 5 PCR primers (black arrowhead) and the expected amplicon sizes are shown next to the strand-specific RT-PCR results. 18S rRNA primers were used in the control reactions. a.u. = arbitrary units. **DOI:** http://dx.doi.org/10.7554/eLife.04106.032

We next interrogated the impact of this set of antisense transcripts. Overall, antisense transcripts showed no effect on mRNA abundance and translational efficiencies of the cognate sense transcript. These observations parallel those described for antisense transcripts in yeast (Brar et al., 2012). Thus, at first approximation antisense transcripts do not appear to play a role in translational regulation. However, these observations could be confounded due to the small number of genes in this set, and we cannot exclude the possibility of sense/anti-sense heterogeneity at the single cell level, obscured here at the population level.

## Discussion

Herein we present, for the first time, a comprehensive view of the coupled transcriptional and translational dynamics of the *P. falciparum* IDC by determination of transcript abundance and architecture together with ribosomal density and positioning. The quality of our data relies on several critical features: (1) high temporal specificity and reproducibility of fully independent biological replicas of five strictly staged cultures; (2) purified merozoites to allow discrete measurements in this stage without confounding contributions from schizonts or rings; (3) monosome isolation from sucrose gradients to specifically enrich for ribosome-derived footprints and avoid potential complications that can arise with methods like sucrose cushions which are prone to mRNA contamination; (4) sufficient sequencing depth of biological replicates to set a statistical threshold for minimum read coverage and to demonstrate reproducibility; (5) stringent strand specificity to facilitate an HMM for the description of transcript boundaries and the detection of antisense transcription.

Previous studies of the transcript abundance in the malaria blood stages revealed a periodic cascade of gene expression, whereby the majority of expressed genes exhibit one peak of expression per cell cycle (Bozdech et al., 2003). The global profile of transcriptional expression was subsequently found to be highly stereotypical across strains and appeared to lack dynamic responses to perturbation (Llinás et al., 2006; Ganesan et al., 2008). It has been suggested that translational control of protein expression could compensate for the lack of transcriptional dynamics. Proteomic studies described delays in peak mRNA and corresponding protein abundance implicating translational or post-translational mechanisms in the modulation of gene expression (Le Roch et al., 2004; Foth et al., 2011).

Our ribosome profiling results reveal a tight coupling of transcription and translation for the majority of expressed genes, indicating that most protein products are translated with highly similar timing and in proportion to their corresponding mRNA transcripts. Synthesized proteins are likely to exert their functions immediately upon translation but post-translational regulation, not captured by our data, could still be at play. Direct correlations of translational efficiencies measured in this study along with proteomic data sets are hampered by the reduced sensitivity of the latter and differences in temporal resolution and staging of the parasites between data sets. However, the available proteomic evidence is largely consistent with the results presented here, particularly for highly translated proteins. The simultaneous capture of mRNA abundance and translation is expected to be a more accurate proxy for protein levels than measurements of mRNA abundance alone (Ingolia et al., 2009) and provides a critical resource for the identification of instances of post-translational regulation of gene expression. However, we note that this data set only provides a direct measure of relative changes in translational efficiencies rather than changes in bulk transcription and translation.

While no up- or down-regulation of global translation efficiencies were observed in any particular stage, more extreme translational efficiencies were measured in subsets of genes expressed in rings and merozoites. We find 177 translationally up-regulated genes with functions predominantly related to merozoite egress and invasion, with peak mRNA in schizonts and peak translational efficiency in rings. It is likely that the genes with unknown functions, regulated in an analogous way during the merozoite to ring transition, are also associated with this process. Our data support a model whereby the transcripts of proteins necessary for merozoite structure and function are made in the previous stage in large abundance, are translationally up-regulated during the invasion process, and remain highly translated well into the ring stage despite rapid mRNA decay during this stage (Shock et al., 2007). Whether the accumulation of 5′ leader ribosome density is a mechanism that assists in this process or is it merely a byproduct of more efficient ribosomal initiation on these templates remains to be tested. With the emergence of genome editing tools such as CRISPR/Cas9 (Ghorbal et al., 2014), it may be possible to create versions of genes with altered cis-acting sequences to test for modulation of 5′ ribosome density and its effect on translational efficiency.

The global nature of ribosome accumulation within the 5′ leader sequences of many transcripts during the IDC and the lack of an association between 5′RD and the number or length of uORFs suggests that ribosomes accumulate on 5′ leaders through means other than a uORF model. For comparison, in yeast under starvation conditions the fraction of ribosome footprints derived from 5′ leaders is increased by sixfold and in some cases no single uORF can account for the entire distribution of ribosomes on the 5′ leader of a gene (Ingolia et al., 2009), much like *P. falciparum*. What mechanism could account for global ribosome accumulation in the 5′ leader? The presence of apparent 80S ribosomes within the 5′ leader sequence, regardless of whether they cover uORFs or not, suggests an engagement mode in which the fidelity of start codon recognition is altered or suspended. Current models propose that the 43S pre-initiation complex loads onto the mRNA with the assistance of other initiation factors near the 5′ cap and proceeds to scan down the length of the mRNA until it encounters an AUG codon. This is followed by the assembly of the 48S preinitiation complex and then finally the 80S complex (for review, see Hinnebusch, 2011). The AUG that is ultimately chosen is not always the first one encountered, and its sequence context is important for recognition. The factors eIF1, eIF1A, and eIF5 have been implicated in recognition of the ‘correct’ AUG (Aitken and Lorsch, 2012). In the case of *P. falciparum*, differential regulation or modification of these factors could plausibly result in altered start codon selection and 80S assembly. Whether prematurely initiated complexes are able to scan without synthesizing a peptide or are required to assemble and reassemble until encountering the right start codon remains an open question. Large 5′ ribosome accumulation on translationally up-regulated genes in the ring stage suggests that premature initiation on these transcripts is not detrimental. The development of an in vitro translation system that recapitulates upstream 80S assembly on *P. falciparum* 5′ leaders will allow direct testing of premature initiation and its effect on translational efficiency in this parasite.

Our ribosome profiling data add an important component to the rich compendium of genome-wide data, including transcript abundance (Bozdech et al., 2003), mRNA decay (Shock et al., 2007), splicing (Sorber et al., 2011), and proteomics for this parasite (Le Roch et al., 2004; Foth et al., 2011). Features such as 5′ leaders, 3′ UTRs, introns, and antisense transcripts are clearly visible and often well delineated. While experimental validation of transcriptional start sites, terminators, and promoters is required, spanning regions between transcripts, such as the one shown in Figure 6, can be used for the search and identification of such functional sites in a reduced sequence space. The data are available at NCBI GEO (accession #GSE58402) to facilitate future queries and normalized read coverage plots for all 5 timepoints are available packed as a single Mochiview file (Caro et al., 2014). Together our results describe a simplified regulatory architecture of gene translation, albeit one that includes peculiar and potentially unique mechanisms specialized for its highly structured and coordinated lifecycle within erythrocytes. Further biochemical dissection of translational initiation mechanisms and determinants of translational efficiency unique to *Plasmodium* may reveal weaknesses that could be exploited for possible therapeutic intervention.

## Materials and methods

### Cell culture

W2 strain cultures were maintained in Hyperflasks (Corning, Corning, NY) in 500 ml RPMIc (RPMI 1640 media supplemented with 0.25% Albumax II (GIBCO, Grand Island, NY), 2 g/L sodium bicarbonate, 0.1 mM hypoxanthine, 25 mM HEPES (pH 7.4), and 50 μg/L gentamycin), at 37°C, 5% O_2_, and 5% CO_2_, to maximum 10–15% parasitemia at 5% hematocrit (HC) and frequent media changes (at least every 6–8 hr). Cells were synchronized by two consecutive sorbitol treatments for three generations, for a total of six treatments. Maximum invasion, point at which half of the culture is either rings or schizonts, was defined as hour zero and independent time points containing ∼10^10^ parasites were harvested 11, 21, 31, and 45 hr later.

### Polysome isolation and library generation

Cultures were incubated for 5 min in 500 ml 37°C RPMIc, 100 µg/ml cycloheximide (Acros Organics, Bridgewater, NJ) and harvested by centrifugation for 8 min at 3.65×*g* at room temperature. An aliquot was removed and flash frozen in liquid nitrogen for total RNA purification, followed by poly(A)-purification and chemical fragmentation with Zn^2+^ to ∼30 nt for consistency in mRNA-Seq library preparation. The remaining culture was treated with ice-cold 0.1% saponin in 1X PBS, 100 µg/ml cycloheximide, for RBC lysis. Parasites were resuspended in ice-cold parasite lysis buffer (15 mM KOAc, 15 mM MgOAc, 10 mM Tris HCl pH 7.4, 0.5 mM DTT, 0.5% Triton X-100, 100 μg/ml cycloheximide) and dripped into a conical tube filled with, and immersed in, liquid nitrogen. Frozen cells transferred placed in liquid nitrogen pre-chilled chambers and pulverized for 3 min at 15 Hz, on a Retsch MM301 mixer mill. Pulverized cells were thawed on ice, and cell debris was removed by centrifugation at 4°C, 16,000×*g* for 10 min. The supernatant was treated with 2.88 U/µg Micrococcal nuclease for 30 min at room temperature and immediately loaded onto sucrose gradients for ultracentrifugation at 35,000 rpm for 3 hr at 4°C in a L8-60 M Beckman centrifuge. Monosome fractions only, were collected to generate ribosome footprint libraries for deep sequencing using the HiSeq 2000 (Illumina, San Diego, CA), as described (Ingolia et al., 2009).

### Merozoite purification

Late stage schizonts (40–44 hpi) were magnetically purified using MACS LD columns (Miltenyi Biotec, San Diego, CA) and resuspended in RPMIc without blood addition. After reaching maximum invasion (1:1 schizont to ring ratio), cultures were harvested by centrifugation at 1500 rpm at room temperature for 5 min. Pelleted cultures were resuspended into fresh RPMIc and placed at 37°C. Merozoites in the supernatant were treated with 100 µg/ml cycloheximide for 5 min at room temperature, harvested at 4000 rpm at 4°C for 5 min and resuspended in RPMIc and passaged again through a MACS LD column. Parasite lysis buffer was added to the merozoite-enriched flow-through and flash frozen in liquid nitrogen. This procedure was repeated three times every 45 min using the original culture. The same procedure described above was used for RNA extraction and library preparation.

### SNP-corrected W2 genome

W2 strain genomic DNA was isolated from >90% synchronized ring stage cultures. Paired end libraries were constructed using the Nextera DNA Sample Prep Kit (Epicenter Biotechnologies, Madison, WI) according to the manufacturer's instructions reducing PCR cycles from nine to six and using 80% A/T dNTPs. Libraries were sequenced using the HiSeq 2000 (Illumina). Reads were aligned to the *P. falciparum* PlasmoDB 3D7 version 7.1 genome using Bowtie 0.12.1 (Langmead et al., 2009) with parameters –v1 –m 1 (one mismatch allowed, unique mapping). A SNP was called when five or more W2 reads supported, with over 90% agreement, a different base than the one found in the *P. falciparum* 3D7 7.1 genome. 19401 SNPs (0.08% of total bases) were detected and used to produce the SNP-corrected W2 genome based on the 3D7 genome. Fastq files are available for download at NCBI SRA, accession #SRP042946.

### Software pipeline, mappability, and rpkM calculation

Quality-filtered ribosome footprints and mRNA sequencing reads were trimmed to remove library adapter sequence, filtered for *P. falciparum* rRNA using blast, and aligned uniquely to the W2 SNP-corrected genome using Bowtie 0.12.1 (Langmead et al., 2009) allowing no mismatches. The percentage mappability was calculated using an *in silico* library of the *P. falciparum* W2 SNP-corrected genome created using a single nucleotide sliding window of 30 nt. The *in silico* library was uniquely aligned to the genome allowing no mismatches. The mappability score is given by the number of 30 nt sequences covering each nt position in the genome, such that any position has a score that ranges from 0 to 30. Both mRNA and ribosome footprint rpkMs were calculated as in Mortazavi et al. (2008), excluding the first 50 bases of each gene to eliminate bias introduced by the observed ribosome accumulation peak near the start codon. Genes with fewer than 80% mappable bases (248 genes) or any overlapping non-CDS feature on the same strand (77 genes) were excluded from this calculation. Data are available for download at NCBI GEO, accession #GSE58402. MochiView genome browser data tracks are available in Supplementary file 1 (Homann et al., 2010).

### Extended phaseogram

The genes of the RNA-seq transcriptome obtained in this work were listed in the same phaseogram order as the previously published microarray transcriptome (Bozdech et al., 2003). The criteria for inclusion of a gene into the phaseogram was mRNA ≥ 32 rM, >2 peak to trough ratios, and Pearson correlation coefficient >0.8 with the expression profiles of the two neighboring genes.

### Hidden Markov model to describe transcript boundaries

The HMM was built using RNA-Seq data obtained in this study and two states: transcript (*t*) or intergenic (*i*) with three possible emissions: (1) read present, (2) read not present but position is unmappable, (3) read not present but the position is mappable. Both state and emission probabilites were calculated using ∼30 kb training set of manually identified transcript and non-transcript regions. The initial probabilities were set to 0.5. Transition probabilities were estimated from the median length of intergenic regions of (1252 nt) and median lengths of CDS regions (2545 nt), where the P_*t->i*_ = (1/2545), P _*t->t*_ = (2544/2545), P_*i->t*_ = (1251/1252), and P_*i->i*_ = (1/1252). We applied the Viterbi algorithm to predict the optimal path of transcript tracks per time point with a 10 nt window resolution. HMM-defined 5′ leader and 3′ UTR coordinates are available for download at NCBI GEO, accession #GSE58402.

### Strand-specific RT-PCR

Total RNA from late stage parasites was isolated and reverse transcribed using SuperScript III (Invitrogen, Carlsbad, CA) according to manufacturer's instructions, using either an antisense-specific primer to Pfs16 (PFD0310w) or a random nonamer. cDNA was amplified using the Pfs16 antisense-specific primer as a forward primer in combination with one of five reverse primers (Supplementary file 3). 18S rRNA primers were used in the control reactions with the random nonamer-derived cDNA.

## Additional files

Supplementary file 1. MochiView genome browser data tracks. **DOI:** http://dx.doi.org/10.7554/eLife.04106.033

Supplementary file 2. RESA2 mapping reads. **DOI:** http://dx.doi.org/10.7554/eLife.04106.034

Supplementary file 3. Strand-specific RT-PCR primers. **DOI:** http://dx.doi.org/10.7554/eLife.04106.035

### Major datasets

The following datasets were generated:

Caro F, Ahyong V, Betegon M, DeRisi JL, 2014, Ribosome Profiling in P. falciparum asexual blood stages, http://www.ncbi.nlm.nih.gov/geo/query/acc.cgi?acc=GSE58402, Publicly available at NCBI Gene Expression Omnibus.

Caro F, Ahyong V, Betegon M, DeRisi JL, 2014, Publicly available at NCBI SRA.

Caro F, Ahyong V, Betegon M, DeRisi JL, 2014, Data from: Genome-wide Regulatory Dynamics of Translation in the Plasmodium falciparum Asexual Blood Stages, http://dx.doi.org/10.5061/dryad.vb855, Available at Dryad Digital Repository under a CC0 Public Domain Dedication.
