# Supplementary material for: Genome-wide regulatory dynamics of translation in the Plasmodium falciparum asexual blood stages
Source: eLife. 2014 Dec 10;3:e04106. doi: 10.7554/eLife.04106 (PMC4371882; doi:10.7554/eLife.04106)
Supplement: Related texts

# Article commentary: Decision letter

**DOI:** 10.7554/eLife.04106.036

**Affiliations:** Cold Spring Harbor Laboratory, United States

eLife posts the editorial decision letter and author response on a selection of the published articles (subject to the approval of the authors). An edited version of the letter sent to the authors after peer review is shown, indicating the substantive concerns or comments; minor concerns are not usually shown. Reviewers have the opportunity to discuss the decision before the letter is sent (see review process). Similarly, the author response typically shows only responses to the major concerns raised by the reviewers.

Thank you for sending your work entitled “Genome-wide Regulatory Dynamics of Translation in the *Plasmodium falciparum* Asexual Blood Stages” for consideration at *eLife*. Your article has been favorably evaluated by Richard Losick (Senior editor) and 3 reviewers, one of whom is a member of our Board of Reviewing Editors.

The Reviewing editor and the other reviewers discussed their comments before we reached this decision, and the Reviewing editor has assembled the following comments to help you prepare a revised submission.

This manuscript presents genome-wide transcriptional maps and association of the detected steady-state RNAs to ribosomes as a means of studying the possible use of translation as a post-transcriptional control mechanism during 5 stages of development in *P. falciparum*. The use of such a regulatory strategy would help to explain why there is a continuous flow of transcription from the majority of genes in *P. falciparum* throughout the development cycle in place of a more regulated and dynamic gene expression strategy. In addition, the authors seek to enhance our understanding of how this regulatory strategy is enacted in this organism. Thus, this is a timely and potentially and important manuscript. However, overall, the manuscript contains several important issues that require attention to help readers better appreciate the messages communicated.

1) Given the critiques of the riboseq approaches (Chew et al, 2013;119 Guttman et al, 2013) it would be important to firmly establish that the RNAs isolated in the ribosome protocol are part of a productive polysome translation complex and instead of individual monosomes. This concern is given support in Figure 1–figure supplement 1 which shows considerable size 80s peaks in the untreated samples for the Late Troph, Schizonts and merozoites stages. In short, the detection of RNA protected by ribosomes in these footprint experiments cannot always be with associated with RNA destined for translation. Wilson & Masel, 2011 have reported that non-productive binding of single ribosomes to mRNA do not undergo productive translation. Thus, RNAseq results obtained from the 80S peak might be compared to RNAseq results obtained from RNAs isolated from the polysome peak. This would act as a control against this problematic possibility.

2) Since the authors do not generate a proteomic dataset for their samples, it is not convincing that the translation efficiency values that they infer from their ribosomal occupancy profiles are direct indicators of protein synthesis. This is especially true given the delay in protein as compared to transcript detection observed during the *P. falciparum* IDC (Le Roch et al., Genome Biol., 2004 and Foth et al., Mol. Cell. Proteomics, 2011). It is also important to note that select noncoding RNAs show a translational efficiency similar to coding regions, even though they are not translated (Ingolia et al., Cell, 2011, and Guttman et al., Cell, 2013); these noncoding RNAs can be distinguished from coding regions only by their ribosomal release score, which was not calculated in this study. Together, the translation efficiency measured here is not enough to indicate if a protein product is being made. The authors have to provide additional evidence or analyses to conclusively state that there is a lack of “overt” translational control.

3) Another reason for the detection of a transcript in the ribosomal occupancy profile could be due to its presence in other ribonucleoprotein complexes that associate or co-sediment with the 80S monosome fractions. The authors infer the same when they discuss the 3' UTRs of transcripts (Figure 9). Could this be the case for other regions of apparent ribosomal occupancy such as the 5' leader sequence? The authors should discuss this in more detail. A straightforward experiment would be to determine the protein composition of the 80S monosome fractions isolated for ribosome profiling in different stages of the IDC. A related question: Is there a lack of polysomes in merozoite stages? This appears to be the case in Figure 1–figure supplement 1.

4) Another concern is that the authors do not discuss their data in light of the polysome occupancy study published by Bunnik et al., Genome Biology, 2013, and the delayed translation phenomenon that has been observed by Le Roch et al., Genome Biol., 2004, and Foth et al., Mol. Cell. Proteomics, 2011.

5) The antisense RNAs section is not 'up-to-date' with the literature. Three recent studies have detected pervasive transcription of the parasite genome – López-Barragán et al., BMC Genomics, 2011; Siegel et al., BMC Genomics, 2014; Wei et al., PLOS One, 2014 – none of which are referenced and discussed here.

6) The authors establish a threshold read count, in terms of reads per million mapped (rM), for downstream analysis. The threshold chosen here seems reasonable for this data set, but I do think there is an important clarification to be made. There are two separate questions that might be addressed by a read count threshold: a minimum expression level for biologically significant effects and a minimum count for statistically reliable analysis. An rM threshold is reasonable for the former, while a raw read count seems more appropriate for the latter. Doubling sequence coverage would permit statistically reliable detection of lower expression levels.

7) At several points the authors discuss a correlation between mRNA abundance and translation, or between transcription and translation; these seem to be used interchangeably. The authors seem to mean that the amount of protein produced correlates well with the abundance of the mRNA that encodes it – but this phrase could also suggest that more abundant mRNAs are translated more highly. The authors should clarify this point when it's first mentioned.

8) The authors discuss relative translational efficiency (TE). Measures of overall translation and TE in different samples are all scaled by the per-million-mapped factor to correct for differences in sequencing depth, etc. This correction obscures changes in the bulk mRNA content or translational activity between different stages. These effects are discussed in e.g. Lovén et al., Cell 2012. The authors state that “Absolute mean translational efficiencies were the same in all stages” but this conclusion is almost an arithmetic identity after applying per-million-mapped normalization. The polysome profiles presented in Figure 1– figure supplement 1 indicate large changes in overall translational activity at different developmental stages.

9) In discussing RESA2 I was confused by Figure 5–figure supplement 2 until I realized that only the truncated reading frame was shown. It would be great to indicate the full-length reading frame on the graph.

10) In discussing the eIF2alpha kinase IK1, the authors note that the gene is translationally silenced, perhaps reflecting one of the few uORF-mediated translational silencing events in Plasmodium. As eIF2alpha phosphorylation can induce a bypass of such silencing, this seems like a potentially interesting auto regulatory system – in animals, the eIF2alpha phosphatase GADD34 is regulated by uORF silencing in order to provide feedback; here it would seem that positive feedback may act instead.

11) The authors discuss qualitatively the observation that many abundant but poorly translated mRNAs are not detectable by proteomics, in contrast to well-translated messages. It would be valuable to quantify detectability, controlling for mRNA abundance, and test less anecdotally whether detectability, as a proxy for protein abundance, follows translation better than mRNA abundance.

12) It is striking that the 5' UTRs in Plasmodium were longer than the 3' UTRs – this differs from the situation in other eukaryotes, over a wide range of absolute UTR length. The authors should comment on this.

# Reply: Author response

**DOI:** 10.7554/eLife.04106.037

*1) Given the critiques of the riboseq approaches (Chew et al, 2013;119* *Guttman et al, 2013) it would be important to firmly establish that the RNAs isolated in the ribosome protocol are part of a productive polysome translation complex and instead of individual monosomes. This concern is given support in* *Figure 1–figure supplement 1* *which shows considerable size 80s peaks in the untreated samples for the Late Troph, Schizonts and merozoites stages. In short, the detection of RNA protected by ribosomes in these footprint experiments cannot always be with associated with RNA destined for translation. Wilson & Masel, 2011 have reported that non-productive binding of single ribosomes to mRNA do not undergo productive translation. Thus, RNAseq results obtained from the 80S peak might be compared to RNAseq results obtained from RNAs isolated from the polysome peak. This would act as a control against this problematic possibility*.

We thank the reviewers for pointing out this important issue. Our response to this issue and #2 are linked; therefore please refer to the section below for our response.

*2) Since the authors do not generate a proteomic dataset for their samples, it is not convincing that the translation efficiency values that they infer from their ribosomal occupancy profiles are direct indicators of protein synthesis. This is especially true given the delay in protein as compared to transcript detection observed during the P. falciparum IDC (Le Roch et al., Genome Biol., 2004 and Foth et al., Mol. Cell. Proteomics, 2011). It is also important to note that select noncoding RNAs show a translational efficiency similar to coding regions, even though they are not translated (Ingolia et al., Cell, 2011, and Guttman et al., Cell, 2013); these noncoding RNAs can be distinguished from coding regions only by their ribosomal release score, which was not calculated in this study. Together, the translation efficiency measured here is not enough to indicate if a protein product is being made. The authors have to provide additional evidence or analyses to conclusively state that there is a lack of “overt” translational control*.

To summarize the questions raised by reviewer points #1 and #2:

A) Do monosome fragments actually represent productive translating ribosomes?

B) Given that there are footprints that apparently map to ncRNAs, which, by definition, are not expected to be translated, what is evidence that the TE values faithfully reflect translational efficiency?

C) What about the Wilson & Masel findings with regard to unproductive ribosome binding?

D) Given that we do not have a proteomic dataset to accompany this study, how do we reconcile our findings with the apparent delay in protein translation that is observed during the IDC as published by Le Roch and Foth?

Issues A & B are inherently linked. First, a number of methods have recently arisen to assist in the discrimination of RNA fragments that derive from ribosome protection as opposed to anything else.

In a recently published study by Ingolia et al. (*Cell Rep*., September 2014), the authors propose a simple metric to distinguish true ribosome footprints from either spurious RNA fragments or non-ribosomal RNA binding proteins by comparing the cumulative read size distribution between protein coding genes and classical non-coding sequences (such as telomerase RNA or RNAse P). According to this study, comparison of read size distribution alone is sufficient to identify true ribosome footprints.

Our manuscript actually uses this exact metric of read size distribution comparisons in Figure S8. Our data clearly shows a difference between footprints mapping to the 5’ leader and CDS, as opposed to noncoding RNA derived fragments (rRNAs, tRNAs). Thus, for each region with sufficient numbers of mapped fragments, we assess whether the cumulative distribution of fragment lengths is consistent with ribosome binding or not.

In another published paper by Guttman et al., the authors use a metric called the ribosomal release score (RRS) which is similar to the disengagement score (DS) from Chew et al, to distinguish true coding regions from noncoding RNAs by determining whether there is evidence for a substantial decrease in the number of ribosome footprints following the stop codon, a feature that would be expected for a legitimately translated region.

We have further analyzed our dataset using the ribosomal release score (RSS) metric for a number of non-coding RNAs for which we have derived TE values and compared these ncRNAs with the rest of our protein coding genes (below). We find that TE values for protein coding genes are well correlated with the ribosome release scores (black dots), yet nearly all ncRNAs yield poor release scores, regardless of TE value (red dots).

**Author response image 1.** 

Thus, both the method using cumulative distributions of fragment lengths and the RRS metric are consistent with ongoing translation in protein coding regions, as opposed to regions within classic ncRNAs (like tRNAs). However, it should be noted that not all regions fit neatly into one category or another. Regions such as the 5’ leader sequences reveal ribosome density with fragment length distributions that are indistinguishable from those within coding regions, yet in such regions, an RRS metric cannot be calculated. Ingolia et al. (Cell Rep., September 2014), argue that translation on transcripts outside of protein-coding regions can in fact produce polypeptides and therefore a TE value is valid for many non-coding sequences such as 5’UTRs or lncRNAs.

Within coding regions, the legitimacy of ribosome protected fragments and their relationship to translational efficiency is now widely supported in a variety of organisms [Stern-Ginossar et al., Science 2012, Oh et al., Cell 2011, Dunn et al., *eLife* 2013, Ingolia et al. Cell 2011, Smith et al., Cell Rep. 2014, reviewed in Ingolia, Nat. Rev Genet. 2014], yet clearly there is still some debate in the field with regard to unexpected ribosome density outside of coding regions. Realizing the complexity of this issue, we have chosen a conservative path for our manuscript, whereby we use TE to only refer to protein coding regions, and RD (ribosomal density) to refer to those outside of the coding regions (like 5’ RD).

C) What about the Wilson & Masel findings with regard to unproductive ribosome binding?

With specific regard to the Wilson & Masel 2011 paper cited by the reviewer, these authors report ribosomal association to stable unannotated transcripts, yet did not actually assess whether these complexes were engaged in actual translation and synthesis of a peptide. In fact they state:

**“**While we do not know the extent to which this ribosomal association leads to translation, these SUTs, apart from RDT1, do not appear to encode functional, protein-coding genes.**”**

The authors speculate that perhaps ribosome association to unannotated or short ORFs may indeed lead to translation “by accident at least at low levels” and “provide raw material for *de novo* birth of protein-coding genes.”

D) How do we reconcile our findings with the apparent delay in protein translation that is observed during the IDC as published by Le Roch and Foth?

With regards to a comparison between the data presented in this manuscript and previous mass spectrometry based studies, the reviewer cites two articles which report a delay in mRNA abundance with respect to protein detection, Le Roch et al., Genome Biol., 2004 and Foth et al., Mol. Cell. Proteomics, 2011.

In Le Roch et al., the authors analyzed a total of 459 mRNA-protein pairs and found that for 171 genes transcript abundance in one stage correlated better with protein levels of the next stage (>0.5 increase in Spearman rank correlation). However, it should be noted that 45 of these 171 transcripts were also better correlated to the proteins in the *previous* stage. By excluding these genes, a delay between transcript abundance and the detection of its corresponding protein may be attributed to only 129 transcripts.

In Foth et al. the authors analyze 125 mRNA-protein pairs and detect multiple isoforms for some of the proteins. A delay between mRNA abundance and protein detection is observed for 63.7% of 237 protein isoforms (it is unclear how many of the 125 genes analyzed this represents).

Here, we report 177 and 124 translationally up- and down-regulated genes, respectively. This represents 295 genes, which is more than twice the number detected in Le Roch or Foth. However, these 295 genes represent only 8% of the 3,605 genes present in our analysis, and for remaining 92%, we find that transcript abundance is proportional to ribosome occupancy throughout the lifecycle.

Thus, while we identify more instances of translational control than all prior studies, these instances appear to be the exception, and not the rule, as the vast majority of genes appear to lack overt translational control as measured by this assay.

*3) Another reason for the detection of a transcript in the ribosomal occupancy profile could be due to its presence in other ribonucleoprotein complexes that associate or co-sediment with the 80S monosome fractions. The authors infer the same when they discuss the 3' UTRs of transcripts (**Figure 9**). Could this be the case for other regions of apparent ribosomal occupancy such as the 5' leader sequence? The authors should discuss this in more detail. A straightforward experiment would be to determine the protein composition of the 80S monosome fractions isolated for ribosome profiling in different stages of the IDC. A related question: Is there a lack of polysomes in merozoite stages? This appears to be the case in* *Figure 1–figure supplement 1*.

The issue of distinguishing between fragments that derive from actual ribosome binding as opposed to other RNA binding proteins is discussed above. In short, we use the cumulative distribution of read lengths in a manner identical to Ingolia et al. (Cell Rep., September 2014), as a discrimination method. Here, we find that the fragments deriving from the 5’ leaders show a perfectly overlapping cumulative distribution to coding regions. This is not the case for known ncRNAs, like tRNAs.

Regardless, whether our 80S fractions contain binding activities other than ribosomes can be assessed by mass spectrometry, as the reviewer points out. During the course of this study, we in fact prepared schizont and merozoite monosome fractions and a pooled schizont polysome fraction for peptide sequencing by tandem mass spectrometry (LC-MS/MS). The results of this analysis are shown below:

**Table:** 

	total spectral counts	% human	% plasmodium	% of plasmodium ribosomal	% of plasmodium non-ribosomal
merozoite monosomes	241	1.7	98.3	100.0	0.0
schizont monosomes	2937	35.5	64.5	89.6	10.4
schizont polysomes	3128	8.7	91.3	95.7	4.3

These results indicate that the monosomes and polysome fractions that we collected contain mostly ribosomal components (89–100%). The remainder 4.3-10.4% of Plasmodium proteins that are non-ribosomal constitute highly expressed proteins such as heat shock proteins, exported proteins, membrane proteins, GAPDH, and other housekeeping genes.

Only 0.26% of spectral counts in the schizont monosome fraction mapped to putative non-ribosomal RNA-binding proteins (such as the DNA/RNA-binding protein Alba). While we cannot exhaustively account for every protein that co-sediments with the 80S peak, this proteomic evidence confirms that the vast majority of the 80S proteins are ribosomal.

With regard to the polysome profiles from merozoites, we agree that the relative peak height of the polysome region compared to the 80S appears reduced. This could be the result of a global reduction in overall translation at this stage. In this study, it should be noted that we do not directly assess bulk or absolute levels of overall translation activity. Rather, this study examines the relative translational efficiency of transcripts on a per-timepoint, per message basis. In merozoites, we do observe a slightly broader distribution of efficiencies, but not dramatically so.

*4) Another concern is that the authors do not discuss their data in light of the polysome occupancy study published by Bunnik et al., Genome Biology, 2013, and the delayed translation phenomenon that has been observed by Le Roch et al., Genome Biol., 2004, and Foth et al., Mol. Cell. Proteomics, 2011*.

For a comparison with Le Roch et al., Genome Biol., 2004, and Foth et al., Mol. Cell. Proteomics, 2011 please see our response to points #1 & #2, part D.

The reviewers ask for a comparison with the work published by Bunnik et al. where polysome-associated mRNA was compared to total steady state mRNA. A major conclusion of this paper is that there exists strong translational control for 471 genes, where the peak of transcriptional abundance is in trophozoite or schizont stage, but they are most highly associated with polysomes in the ring stage.

There are a number of confounding factors that obstruct a fair comparison on the work presented here and that of Bunnik et al. These factors include the strain, the time points sampled, the methodology for isolation of polysomes, and most importantly, the overall sequencing coverage. We discuss each of these factors below.

In Bunnik et al., the authors used the 3D7 strain of *P. falciparum* to assess hours 0, 18, and 36 hours post invasion whereas we used the W2 strain to sample hours 11, 21, 31, 45 in addition to purified merozoites.

A critical step in ribosome profiling is the isolation of intact polysomes. In our hands, directly using sucrose gradients yields polysome profiles enriched in higher order polysomes, with more than 3 ribosomes. Polysome profiles in Bunnik et al. are obtained by pelleting parasite lysates on sucrose cushions, and re-loading the ribosomal pellets on sucrose gradients. This additional step may cause the dissociation of ribosomes from mRNAs, and could explain the reduced amount of higher order polysomes in their profiles, which in turn may skew their assessment of translation towards lower values. (Compare Bunnik Figure 1 A-D)

Finally, sequencing coverage and biological replicates are the most important determinant of obtaining reliable data in our experiments. In Bunnik et al, there is only a single biological replicate (just the schizont stage). Their deepest exome-wide coverage for polysomes is 12.7x (Table 1, Bunnik et al), and their average coverage for polysomes is 5.3x. The average exome-wide coverage of our ribosome footprint data is 159.9x, which is, on average, 30 times greater than Bunnik et al. (table below).

**Table:** 

	X-fold exome-wide coverage
timepoint	ribosome footprints
rings	315.3
early troph.	205.8
late troph.	55.3
schizonts	53.6
merozoites	169.7
**average**	**159.9**
**average all**	**137.4**

More importantly, the major difference between the Bunnik dataset and our own is the set of 471 genes that the authors identify as being translationally up-regulated in rings. Approximately 76% of these genes fall below our threshold for minimum read coverage (32rM) for analysis, despite the fact that our ring stage coverage is 456-fold greater than the Bunnik zero hour polysome sample (315x coverage compared to Bunnik’s 0.69x).

Of the remaining genes (excluding apicoplast genes, var, rifin, and stevors), we identified only five genes that we would consider to be translationally up-regulated in rings.

Through our own biological replicates of each time point and our assessment of reliability with respect to sequencing coverage (Figure 1–figure supplement 3), we believe it would be inappropriate to draw conclusions for genes or whole datasets that lack sufficient coverage, and thus it is not possible to cleanly compare the two datasets.

*5) The antisense RNAs section is not 'up-to-date' with the literature. Three recent studies have detected pervasive transcription of the parasite genome – López-Barragán et al., BMC Genomics, 2011; Siegel et al., BMC Genomics, 2014; Wei et al., PLOS One, 2014 – none of which are referenced and discussed here*.

We thank the reviewers for pointing this out. We updated our antisense section to reference López-Barragán et al., BMC Genomics, 2011 and Siegel et al., BMC Genomics, 2014. The text now reads “Previous serial analysis of gene expression (SAGE) (Patankar et al. 2001), nuclear run-on experiments (Militello et al. 2005) and more recently antisense splicing events detected by RNA-seq (Sorber et al. 2011; López-Barragán et al. 2011), suggest that antisense RNAs are synthesized by RNA pol II and may constitute up to ∼12% of the erythrocytic-stage steady-state RNA (Gunasekera et al. 2004), yet their presence and biological role, if any, remains unclear. A more recent study found no correlation between natural antisense transcript levels and protein abundance (Siegel et al. 2014).”

The work by Wei et al. analyzes uncapped/non-adenylated intermediate size ncRNAs, which we do not focus on.

*6) The authors establish a threshold read count, in terms of reads per million mapped (rM), for downstream analysis. The threshold chosen here seems reasonable for this data set, but I do think there is an important clarification to be made. There are two separate questions that might be addressed by a read count threshold: a minimum expression level for biologically significant effects and a minimum count for statistically reliable analysis. An rM threshold is reasonable for the former, while a raw read count seems more appropriate for the latter. Doubling sequence coverage would permit statistically reliable detection of lower expression levels*.

We took a conservative approach by choosing a minimum of rM of 32 per gene. We agree with the reviewer that a reliable statistical analysis could still be obtained by lowering this threshold and we did so for the antisense analysis where we chose a ≥16rM bar. Future studies into these low count reads will be necessary to establish their biological significance.

*7) At several points the authors discuss a correlation between mRNA abundance and translation, or between transcription and translation; these seem to be used interchangeably. The authors seem to mean that the amount of protein produced correlates well with the abundance of the mRNA that encodes it – but this phrase could also suggest that more abundant mRNAs are translated more highly. The authors should clarify this point when it's first mentioned*.

We fully agree with the reviewers and clarified the ambiguity by changing the text from “To determine the exact level of correlation between mRNA abundance and translation we directly compared mRNA and ribosome footprint density measurements (Figure 2C). In general, translation is tightly correlated with transcription for all phasic and non-phasic genes in rings (r = 0.85), early trophozoites (r = 0.93), late trophozoites (r = 0.91), schizonts (r = 0.89) and purified merozoites (r = 0.86)”, to “To determine the exact level of correlation between transcription and translation we directly compared mRNA and ribosome footprint density measurements (Figure 2C). In general, translation is tightly correlated with transcription for all phasic and non-phasic genes in rings (r = 0.85), early trophozoites (r = 0.93), late trophozoites (r = 0.91), schizonts (r = 0.89) and purified merozoites (r = 0.86). This indicates that when an mRNA is detected in one stage it is associated proportionally with ribosomes within the same stage.”

We changed: “The simultaneous capture of transcription and translation is expected to be…” to “The simultaneous capture of mRNA abundance and translation is expected to be…”

We changed the phrase “… peak transcription occurring in rings and schizonts” to “…peak mRNA abundance occurring in rings and schizonts”.

*8) The authors discuss relative translational efficiency (TE). Measures of overall translation and TE in different samples are all scaled by the per-million-mapped factor to correct for differences in sequencing depth, etc. This correction obscures changes in the bulk mRNA content or translational activity between different stages. These effects are discussed in e.g. Lovén et al., Cell 2012. The authors state that “Absolute mean translational efficiencies were the same in all stages” but this conclusion is almost an arithmetic identity after applying per-million-mapped normalization. The polysome profiles presented in* *Figure 1–figure supplement 1* *indicate large changes in overall translational activity at different developmental stages*.

This study measures the relative change in translational efficiency on a per-gene, per-time point basis, and does not measure absolute changes in bulk translation or transcription, as the reviewer correctly points out. To clarify this for the reader, we have added a statement in the Discussion highlighting this important point “However, we note that this dataset only provides a direct measure of relative changes in translational efficiencies rather than changes in bulk transcription and translation.”

With respect to the statement that the reviewer highlights regarding mean translational efficiencies, we have corrected and amended this sentence, since the mean TEs are in fact not identical in all stages (for example, the mean TE in schizonts compared to early trophs are 1.47-fold different). As shown in Figure 4, the shape of the TE distributions are not identical, and thus we would indeed expect some variation in the mean.

The text now reads:

“Absolute mean translational efficiencies in all stages (log_2_TE *µ*_Rings_ = -0.43, log_2_TE *µ*_E.trophs._ = -0.56, log_2_TE *µ*_L.trophs._ = -0.31, log_2_TE *µ*_Schizonts_ = -0.16 and log_2_TE *µ*_Merozoites_ = -0.68) had a maximum fold difference of 1.47-fold observed between early trophozoites and schizonts.”

9) In discussing RESA2 I was confused by *Figure 5–figure supplement 2* until I realized that only the truncated reading frame was shown. It would be great to indicate the full-length reading frame on the graph.

We thank the reviewers for indicating to us how to make the figure clearer. As advised, we added the annotated gene model described in PlasmoDB version 7.1, which is the version used for reference in this work.

**Author response image 2.** 

*10) In discussing the eIF2alpha kinase IK1, the authors note that the gene is translationally silenced, perhaps reflecting one of the few uORF-mediated translational silencing events in Plasmodium. As eIF2alpha phosphorylation can induce a bypass of such silencing, this seems like a potentially interesting auto regulatory system – in animals, the eIF2alpha phosphatase GADD34 is regulated by uORF silencing in order to provide feedback; here it would seem that positive feedback may act instead*.

We fully agree with the reviewers. Ribosome footprints partially overlap with 2 of the 18 uORFs counted on the 5’ leader of the eIF2 alpha kinase transcript. Whether these uORFs or other 5’ leader motifs are responsible for the silencing of the downstream coding region remains an important question. Additionally, this gene presents the lowest ribosomal release score calculated, which strongly suggests that it is not being translated.

*11) The authors discuss qualitatively the observation that many abundant but poorly translated mRNAs are not detectable by proteomics, in contrast to well-translated messages. It would be valuable to quantify detectability, controlling for mRNA abundance, and test less anecdotally whether detectability, as a proxy for protein abundance, follows translation better than mRNA abundance*.

We did not wish to make the statement that genes that have high mRNA abundance would not be detected by proteomic methods, and we thank reviewers for highlighting this misunderstanding. We have therefore revised our manuscript as following:

We changed the phrase from: “Thus, our ribosome profiling dataset highlights instances of translational control of genes for which, in the presence of abundant mRNA, the protein counterpart is unlikely to be detected by proteomic methods” to “Thus, our ribosome profiling dataset highlights instances of translational control of genes that may not be detected by proteomic methods.”

*12) It is striking that the 5' UTRs in Plasmodium were longer than the 3' UTRs – this differs from the situation in other eukaryotes, over a wide range of absolute UTR length. The authors should comment on this*.

A review of the current literature reveals a wide distribution of different 5’UTR sizes in different types of organisms. Previous studies found that 5’UTRs of mRNAs encoding genes which need to be strongly and finely regulated are often longer than average (Kozak 1987; Hurowitz and Brown 2003; David et al. 2006; Nagalakshmi et al. 2008; Bruno et al. 2010). On the other hand, Lynch et al. (2005) proposed that the length of the 5’UTR is driven mainly by random genetic drift and mutational processes that cause stochastic turnover in transcription-initiation sites and premature start codons, combined with a selective constrain against the premature initiation of translation. Additional sequencing and mapping of transcript architecture in other eukaryotes will help establish in the future whether or not *P. falciparum* truly lies at the extreme of the distribution.
